# Genetic susceptibility and lifestyle modify the association of long-term air pollution exposure on major depressive disorder: a prospective study in UK Biobank

**DOI:** 10.1186/s12916-023-02783-0

**Published:** 2023-02-21

**Authors:** Dankang Li, Junqing Xie, Lulin Wang, Yu Sun, Yonghua Hu, Yaohua Tian

**Affiliations:** 1 Ministry of Education Key Laboratory of Environment and Health, and State Key Laboratory of Environmental Health (Incubating), School of Public Health, Tongji Medical College, Huazhong University of Science and Technology, No.13 Hangkong Road, Wuhan, 430030 China; 2 Department of Maternal and Child Health, School of Public Health, Tongji Medical College, Huazhong University of Science and Technology, No.13 Hangkong Road, Wuhan, 430030 China; 3 Center for Statistics in Medicine, NDORMS, University of Oxford, The Botnar Research Centre, Oxford, UK; 4 Department of Otorhinolaryngology, Union Hospital, Tongji Medical College, Huazhong University of Science and Technology, 1277 Jiefang Avenue, Wuhan, 430022 China; 5 Department of Epidemiology and Biostatistics, School of Public Health, Peking University, No.38 Xueyuan Road, Beijing, 100191 China

**Keywords:** Air pollution, Major depressive disorder, Genetic susceptibility, Healthy lifestyle, Interaction

## Abstract

**Background:**

Evidence linking air pollution to major depressive disorder (MDD) remains sparse and results are heterogeneous. In addition, the evidence about the interaction and joint associations of genetic risk and lifestyle with air pollution on incident MDD risk remains unclear. We aimed to examine the association of various air pollutants with the risk of incident MDD and assessed whether genetic susceptibility and lifestyle influence the associations.

**Methods:**

This population-based prospective cohort study analyzed data collected between March 2006 and October 2010 from 354,897 participants aged 37 to 73 years from the UK Biobank. Annual average concentrations of PM_2.5_, PM_10_, NO_2_, and NO_x_ were estimated using a Land Use Regression model. A lifestyle score was determined based on a combination of smoking, alcohol drinking, physical activity, television viewing time, sleep duration, and diet. A polygenic risk score (PRS) was defined using 17 MDD-associated genetic loci.

**Results:**

During a median follow-up of 9.7 years (3,427,084 person-years), 14,710 incident MDD events were ascertained. PM_2.5_ (HR: 1.16, 95% CI: 1.07–1.26; per 5 μg/m^3^) and NO_x_ (HR: 1.02, 95% CI: 1.01–1.05; per 20 μg/m^3^) were associated with increased risk of MDD. There was a significant interaction between the genetic susceptibility and air pollution for MDD (*P*-interaction < 0.05). Compared with participants with low genetic risk and low air pollution, those with high genetic risk and high PM_2.5_ exposure had the highest risk of incident MDD (PM_2.5_: HR: 1.34, 95% CI: 1.23–1.46). We also observed an interaction between PM_2.5_ exposure and unhealthy lifestyle (*P*-interaction < 0.05). Participants with the least healthy lifestyle and high air pollution exposures had the highest MDD risk when compared to those with the most healthy lifestyle and low air pollution (PM_2.5_: HR: 2.22, 95% CI: 1.92–2.58; PM_10_: HR: 2.09, 95% CI: 1.78–2.45; NO_2_: HR: 2.11, 95% CI: 1.82–2.46; NO_x_: HR: 2.28, 95% CI: 1.97–2.64).

**Conclusions:**

Long-term exposure to air pollution is associated with MDD risk. Identifying individuals with high genetic risk and developing healthy lifestyle for reducing the harm of air pollution to public mental health.

**Supplementary Information:**

The online version contains supplementary material available at 10.1186/s12916-023-02783-0.

## Background

Major depressive disorder (MDD) is among the most common mental illnesses, and it severely limits psychosocial functioning and negatively affects the quality of life [1]. MDD affects approximately 6% of the adult population worldwide each year [2], and patients with MDD are nearly 20-fold more likely to die by suicide than individuals without MDD [3]. According to the World Health Organization, MDD will be the leading disease burden worldwide by 2030 [4].

Evidence indicates that socioeconomic status, medical conditions, and family history play a major role in the development of mental health disorders and that environmental factors may also influence the development of such disorders through neuroinflammatory pathways and oxidative stress [5, 6]. Although air pollution is the most common environmental risk to human health, results on the correlation between air pollution and health are sparse and inconsistent. For example, a recent systematic review reported that long-term exposure to air pollution was associated with an increased risk of depression, but the association was not significant in more than half of the studies included in the review [7]. Furthermore, the size and quality of these studies varied considerably. Therefore, additional large population-based cohort studies are necessary to test the potential association between long-term exposure to air pollution and MDD.

Evidence suggests that genetic factors play a critical role in the development of MDD [8, 9]. A genome-wide association study (GWAS) identified some genetic variants associated with MDD risk [10]. Analyzing the cumulative genetic burden of these genetic variants by using polygenic risk scores (PRSs) could provide quantitative measures of genetic susceptibility and could help effectively identify individuals at high risk of MDD [11]. Recent studies have suggested that genetic susceptibility may influence the environment–disease relationship [12, 13]. However, the influence of genetic susceptibility on the association between exposure to air pollution and MDD risk is unclear. In addition, unhealthy lifestyle behaviors, such as smoking, excessive alcohol intake, and lack of physical activity, increase the risk of MDD [14–16]. Several studies have revealed that healthy lifestyle habits played a pivotal role in attenuating the effect of air pollution on the risk of various diseases [17–19]. However, whether a healthy lifestyle can mitigate the effect of air pollution on MDD risk is unclear.

To address the aforementioned questions, this study investigated the association between exposure to air pollution and the incidence of MDD in a large population-based cohort (UK Biobank). In addition, we examined the potential modifying effect of genetic susceptibility and lifestyle on this association.

## Methods

### Study population

In this prospective cohort study, we sourced data from the UK Biobank. Details of the design and survey methods of the UK Biobank have been described elsewhere [20]. Briefly, the baseline survey was done between March 13, 2006, and October 1, 2010, at 22 assessment centers in urban areas of England, Wales, and Scotland [20]. More than 0.5 million participants provided demographics, socioeconomics, lifestyle, and health information through touchscreen questionnaires and anthropometric measurements [21].

In the present study, we excluded participants who had experienced a MDD at baseline (*N* = 31,325) and those with MDD identified by medical records at baseline from the current study (*N* = 9278). Then, we excluded participants with missing information on genetic data (*N* = 72,717) and air pollution (*N* = 34,263). Finally, data from 354,897 individuals were available for the final analyses (Additional file 1: Fig. S1). All participants provided informed consent to participate.

The authors assert that all procedures contributing to this work comply with the ethical standards of the relevant national and institutional committees on human experimentation and with the Helsinki Declaration of 1975, as revised in 2008. All procedures involving human subjects/patients were approved by the North West Multi-centre Research Ethics Committee (REC reference: 16/NW/0274).

### Air pollutants

Estimates of particulate matter with aerodynamic diameter ≤ 2.5μm (PM_2.5_), particulate matter with aerodynamic diameter ≤ 10μm (PM_10_), nitrogen dioxide (NO_2_), and nitrogen oxides (NO_x_) concentrations were available for the year 2010. Land Use Regression (LUR) techniques were employed to model the annual average concentrations of these air pollutants by using the predictor variables obtained from the Geographic Information System such as traffic, land use, and topography [22, 23]. Air pollution exposures for all participants in the UK Biobank were linked to the records through geocoded residential addresses given at the baseline visit. LUR techniques were developed by the ESCAPE project, which estimates for particulates are valid up to 400 km from the monitoring area and required a spatial resolution of at least 100 m. More details of the ESCAPE LUR model development and validation methodology have been published elsewhere. Briefly, leave-one-out cross-validation showed good model performance for PM_2.5_, PM_10_, NO_2_, and NO_x_ (cross-validation *R*^2^=77%, 88%, 87%, and 88%, respectively) [23].

### Polygenic risk score

We used the imputed genetic data from UK Biobank. Details of genotyping, imputation, and quality control have been described previously [24]. In the present analysis, 17 single-nucleotide polymorphisms (SNPs) were selected based on their association with MDD in previous GWAS to create a weighted PRS for MDD (selected SNPs are provided in Additional file 1: Table S1) [10]. Details regarding PRS calculation have been described in a previous study [12]. We calculated PRS by summing the product of the weighted risk estimate (the SNP’s *β* coefficient from reported MDD GWAS) and the number of risk alleles (0, 1, and 2) for each risk variant: PRS = ${\sum}_{n=1}^M{\beta}_n\times {\textrm{SNP}}_n$. In the present study, subjects were categorized as low, intermediate, and high genetic risk based on the tertile distribution of PRS.

### Lifestyle score

Consistent with the previous study, the lifestyle score was constructed based on the following lifestyle variables: smoking status, alcohol intake, physical activity, television viewing time, sleep duration, fruit and vegetable intake, oily fish intake, red meat intake, and processed meat intake [25]. These factors comprise the score derived from touchscreen questionnaire responses at baseline (Additional file 1: Table S2). Participants scored 1 point for each unhealthy category defined on the basis of national guidelines (Additional file 1: Table S2). Unhealthy lifestyle was assessed as follows: current smoker, alcohol consumed daily or almost daily, <150 min per week of moderate-intensity physical activity or <75 min per week of vigorous-intensity physical activity, ≥4 h per day of television viewing time, <7 h or >9 h of sleep time per day, <400 g of fruits and vegetables per day, <1 portion of oily fish per week, >3 portions of red meat per week, and >1 portion of processed meat per week. An unweighted score was created by summing each component score with a range from 0 to 9, with higher scores indicating an unhealthier lifestyle. Then, participants were subsequently categorized into three categories: most healthy (scored 0, 1, or 2), moderately healthy (scored 3, 4, or 5), and least healthy (scored 6, 7, 8, or 9) [25].

### Assessment of MDD

Incident outcomes in the UK Biobank were linked to hospital admissions and death registries. Detailed information has been described elsewhere [26]. Incident MDD was diagnosed using the International Classification of Diseases, Tenth Revision (ICD-10) coding system and the ICD-10 codes of the categories of disorders are shown in Additional file 1: Table S3. Participants were followed from January 1, 2011, to the first diagnosis of incident MDD, death, or until January 30, 2021, whichever came first (the timeline is shown in Additional file 1: Fig. S2).

In line with previous studies [27], individuals who experienced a prevalent MDD at baseline were defined as per UK Biobank’s assessment protocol for lifetime experience of probable MDD. The structured and validated diagnostic criteria were used to assess the lifetime history of mood disorders in the UK Biobank [28, 29]. To briefly summarize, the assessment of MDD comprised a series of items from the Patient Health Questionnaire, items relating to lifetime experience of minor or major depression, and items on social support for mental health [28, 30]. The criteria for participants who had experienced a MDD included those who had experienced a single probable lifetime episode of major depression, probable recurrent major depression (moderate), or probable recurrent major depression (severe), or any combination thereof (panel).

### Covariates

We developed a directed acyclic graph (DAG) to identify potential covariates that need to be adjusted in our multivariate analyses, using the online DAGitty tool (www.dagitty.net) [31]. Based on the priori knowledge and existing literature, we included a rich set of covariates in the DAG that should be considered in the analyses [27, 32, 33]. From the DAG (Additional file 1: Fig. S3), a minimally sufficient set of variables for adjustment were retained: age; gender (female or male); ethnicity (white; mixed; Asian; black; Chinese; or other); education level (college or university degree; A/AS levels or equivalent; O level/GCSE or equivalent; CSE or equivalent; NVQ or HND or HNC or equivalent; other professional qualification; or none of the above); employment status (employed; retired; or unemployed, home maker, or others); household income (less than £31,000 or £31,000 and above); and Townsend deprivation index (continuous). The proportions of missing data about covariates were as follows: 15% for household income, 2% for education level and employment status, and less than 1% for ethnicity and Townsend deprivation index. Multiple imputation by fully conditional specification (FCS) was performed to impute missing covariate data.

### Statistical analyses

Continuous and categorical variables were presented as mean ± standard deviation (SD) and number (percentage), respectively. Cox proportional hazard regression models were used to estimate the associations between ambient air pollutants, genetic risk, and lifestyle score with incident MDD and to calculate the hazard ratio (HR) and 95% confidence interval (CI). Schoenfeld residuals were used to test the assumptions of proportionality (Additional file 1: Fig. S4-S7). The Cox regression model was unadjusted in model 1. Model 2 was adjusted for age, gender, ethnicity, education, employment status, household income, and Townsend deprivation index. In addition, we then conducted several sensitivity analyses to assess the robustness of the findings. First, we excluded participants with MDD that occurred during the first 2 years of follow-up to minimize the influence of reverse causation. Second, we restricted all analyses among participants who resided in their current address for 5 or more years to consider the effects of reliable accumulated exposures. Third, to avoid misclassification bias and potential confounding, we excluded participants who were diagnosed with dementia (ICD-10 codes G30.x, G31.x, and F00.x–F03) or anxiety disorders (ICD-10 codes F40.x and F41.x) during the follow-up period. Fourth, we repeated the analyses by additional adjusting for BMI categories, cardiometabolic disease, diabetes, lifestyle, and MDD-PRS to reduce potential residual confounding. Fifth, we restricted the analysis to participants with complete covariate data for comparison with the results based on multiple imputation. Sixth, we examined the association between time-varying exposure to air pollution and MDD risk. Exposures to PM_2.5_, PM_10_, NO_2_, and NO_x_ during the follow-up were estimated at each participant’s residential addresses using data from the UK’s Department for Environment, Food and Rural Affairs based on a previous study [34]. Finally, to ensure comprehensive ascertainment, incident MDD was derived from linkage to both hospital inpatient and primary care records. In addition, to investigate the effects of pollutant mixtures and eliminate the problem of multicollinearity, principal component analysis (PCA) was applied [35].

We examined the dose–response association between air pollutants and MDD risk using restricted cubic spline regressions. We additionally examined the combination of air pollutants and genetic categories with incident MDD risk (12 categories with lowest genetic risk and lowest quartile of air pollution as reference) and the combination of air pollutants and lifestyle with incident MDD (12 categories with lowest lifestyle risk and lowest quartile of air pollution as reference). Moreover, the interactions between genetic risk and lifestyle with air pollutants were tested by stratifying genetic risk and lifestyle.

All data were analyzed using R (version 4.0.5) and the statistical significance was set to *P* value < 0.05 at two tails.

## Results

During a total of 3,427,084 person-years (median follow-up 9.7 years), 14,710 incident MDD events were recorded. Table 1 displays the baseline characteristics of the study participants. Participants who developed MDD were more likely to have the least healthy lifestyle, high genetic risk, and higher air pollution exposure. The Pearson correlation coefficients and the summary statistics of air pollutants are shown in Additional file 1: Fig. S8 and Additional file 1: Table S4, respectively.

**Table 1:** Descriptive characteristics of the study participants and stratified by major depressive disorder (MDD) status at follow-up

Variables	Total (*n*=354,897)	Individuals without MDD (*n*=340,187)	Individuals with MDD (*n*=14,710)
Age, years (mean ± SD)	56.71 ± 8.07	56.71 ± 8.07	56.87 ± 8.08
Gender, *n* (%)			
Female	189,358 (53.4)	179,981 (52.9)	9377 (63.7)
Male	165,539 (46.6)	160,206 (47.1)	5333 (36.3)
Ethnicity, *n* (%)			
White ethnicity	340,408 (96.3)	326,236 (96.3)	14,172 (96.9)
Mixed ethnicity	1600 (0.5)	1519 (0.4)	81 (0.6)
Asian ethnicity	5131 (1.5)	4966 (1.5)	165 (1.1)
Black ethnicity	3105 (0.9)	3014 (0.9)	91 (0.6)
Chinese ethnicity	783 (0.2)	775 (0.2)	8 (0.1)
Other ethnicities	2322 (0.7)	2214 (0.7)	108 (0.7)
Missing data	1548 (0.4)	1463 (0.4)	85 (0.6)
Education level, *n* (%)			
College or university degree	111,478 (31.8)	108,187 (32.2)	3291 (22.8)
A/AS levels or equivalent	39,041 (11.1)	37,616 (11.2)	1425 (9.9)
O level/GCSE or equivalent	77,115 (22.0)	73,973 (22.0)	3142 (21.8)
CSE or equivalent	20,170 (5.8)	19,148 (5.7)	1022 (7.1)
NVQ or HND or HNC or equivalent	23,484 (6.7)	22,381 (6.7)	1103 (7.6)
Other professional qualifications	18,141 (5.2)	17,385 (5.2)	756 (5.2)
None of the above	60,991 (17.4)	57,287 (17.1)	3704 (25.6)
Missing data	4477 (1.3)	4210 (1.2)	267 (1.8)
Employment status, *n* (%)			
Employed	203,737 (58.0)	197,160 (58.5)	6577 (45.3)
Retired	120,405 (34.3)	115,095 (34.2)	5310 (36.6)
Unemployed, home maker, or others	27,126 (7.7)	24,506 (7.3)	2620 (18.1)
Missing data	3629 (1.0)	3426 (1.0)	203 (1.4)
Household income, *n* (%)			
Less than £31,000	143,987 (47.7)	136,113 (46.9)	7874 (65.3)
£31,000 and above	157,996 (52.3)	153,806 (53.1)	4190 (34.7)
Missing data	52,914 (14.9)	50,268 (14.8)	2646 (18.0)
Townsend deprivation index, (mean ± SD)	−1.46 ± 2.98	−1.50 ± 2.96	−0.65 ± 3.35
Missing data	369 (0.1)	360 (0.11)	9 (0.06)
Healthy lifestyle factors, *n* (%)			
Smoking	34,974 (9.9)	32,459 (9.6)	2515 (17.2)
Alcohol intake	74,074 (20.9)	71,391 (21.0)	2683 (18.3)
Physical activity	55,686 (19.5)	52,823 (19.2)	2863 (26.1)
TV viewing	102,308 (28.9)	96,759 (28.5)	5549 (37.8)
Sleep time	93,612 (26.4)	88,292 (26.0)	5320 (36.2)
Fruit and vegetable intake	63,882 (18.5)	60,721 (18.4)	3161 (22.5)
Oily fish intake	156,756 (44.2)	149,735 (44.1)	7021 (47.8)
Red meat intake	174,118 (49.7)	167,380 (49.8)	6738 (46.8)
Processed meat intake	112,094 (31.6)	107,355 (31.6)	4739 (32.3)
Lifestyle category, *n* (%)			
More healthy	152,734 (54.7)	147,875 (55.1)	4859 (46.2)
Moderately healthy	117,865 (42.2)	112,779 (42.0)	5086 (48.3)
Least healthy	8489 (3.0)	7907 (2.9)	582 (5.5)
Genetic risk category, *n* (%)			
Low	118,478 (33.4)	113,888 (33.5)	4590 (31.2)
Intermediate	118,295 (33.3)	113,420 (33.3)	4875 (33.1)
High	118,124 (33.3)	112,879 (33.2)	5245 (35.7)
Air pollution, μg/m^3^ (mean ± SD)			
PM_2.5_	9.98 ± 1.06	9.97 ± 1.06	10.13 ± 1.09
PM_10_	16.20 ± 1.90	16.20 ± 1.91	16.30 ± 1.88
NO_2_	26.41 ± 7.59	26.37 ± 7.58	27.22 ± 7.65
NO_X_	43.68 ± 15.55	43.60 ± 15.52	45.53 ± 16.05

Continues variables are displayed as means ± SD, and categorical variables are displayed as numbers (percentages)

*Abbreviations*: *SD* standard deviation, *A/AS* advanced, *CSE* Certificate of Secondary Education, *GCSE* General Certificate of Secondary Education, *HNC* Higher National Certificate, *HND* Higher National Diploma, *NVQ* National Vocational Qualification, *BMI* body mass index, *MDD* major depressive disorder, *PM*_*2.5*_ fine particulate matter with diameter ≤2.5μm, *PM*_*10*_ particulate matter with diameter ≤10μm, *NO*_*2*_ nitrogen dioxide, *NO*_*X*_ nitrogen oxides

**Fig. 1:**
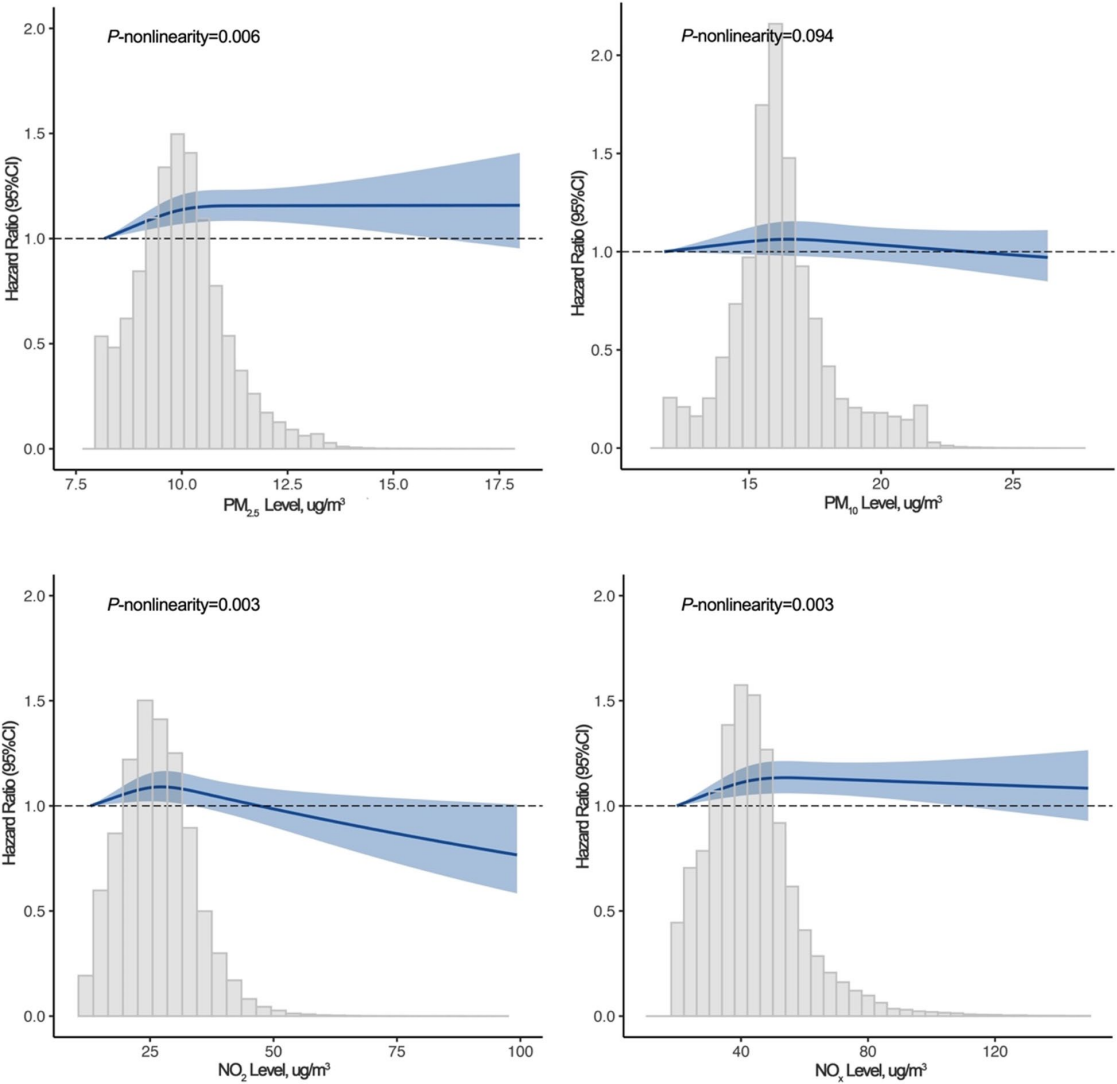
Dose–response relationship of long-term exposure to air pollution and incident major depressive disorder (MDD). Multiple-adjusted hazard ratio (HR) for continuous air pollution is modeled using restricted cubic splines. Models are adjusted for age, gender, ethnicity, education level, employment status, household income, and Townsend deprivation index. The reference group is considered the minimum exposure level of air pollution in the entire population. Gray bars represent the distribution of the exposure levels in the entire population. The blue solid line indicates HR and the shaded area indicates a 95% confidence interval

Table 2 presents the relations of each air pollutant with MDD risk. In the finally multivariate-adjusted model, PM_2.5_ (HR: 1.16, 95% CI: 1.07–1.26; per 5 μg/m^3^) and NO_x_ (HR: 1.02, 95% CI: 1.01–1.05; per 20 μg/m^3^) were significantly associated with MDD. However, we did not observe an association between PM_10_ (HR: 1.00, 95% CI: 0.92–1.09; per 10 μg/m^3^) and NO_2_ (HR: 1.00, 95% CI: 0.98–1.02; per 10 μg/m^3^) with MDD. Moreover, compared with the lowest quartile, the HRs (95% CIs) of MDD were 1.12 (1.06, 1.17) and 1.07 (1.02, 1.13) for subjects with the highest quartile of exposure to PM_2.5_ and NO_x_, respectively. These results did not alter appreciably in the sensitivity analyses (Additional file 1: Table S5-S12). We then examined the association between principal components (PCs) and MDD risk (Additional file 1: Table S13). We found that the first PC (PC1) predominated by PM_2.5_, NO_2_, and NO_x_ was associated with an increased risk of MDD (HR = 1.02, 95% CI: 1.01, 1.04). However, no significant association was observed for PC2 which predominated by PM_10_ (HR = 1.00, 95% CI: 0.98, 1.01). We also used the restricted cubic spline to assess the potential dose-response relationship of air pollutants with MDD in Fig. 1. We found nonlinear relationships between PM_2.5_, NO_2_, and NO_x_ with the risk of incident MDD (*P*-nonlinearity for PM_2.5_: 0.006; NO_2_: 0.003; NO_x_: 0.003). Additional file 1: Table S14 shows results about the association between air pollution and MDD stratified by age and sex group. No significant interaction was found between air pollution and age on the risk of incident MDD, whereas significant interactions were observed between PM_2.5_ with gender on the risk of incident MDD (*P*-interactions were 0.049 for PM_2.5_). The association of air pollution exposure with MDD risk was stronger in men than in women (Additional file 1: Table S14).

**Table 2:** Association between long-term exposure to air pollutants and major depressive disorder (MDD)

Air pollution	No. MDD cases/person-years	MDD HR (95% CI)	
		Model 1	Model 2
PM_2.5_, per 5-μg/m^3^ increase	--	1.92 (1.79, 2.07)	1.16 (1.07, 1.26)
Q1	3075/880,978	1.00 (Ref.)	1.00 (Ref.)
Q2	3452/849,441	1.16 (1.11, 1.22)	1.08 (1.03, 1.13)
Q3	3885/858,381	1.30 (1.24, 1.36)	1.12 (1.06, 1.17)
Q4	4398/838,285	1.47 (1.40, 1.54)	1.11 (1.06, 1.17)
*P* for trend		<0.001	<0.001
PM_10_, per 10-μg/m^3^ increase	--	1.30 (1.20, 1.41)	1.00 (0.92, 1.09)
Q1	3420/879,257	1.00 (Ref.)	1.00 (Ref.)
Q2	3741/868,525	1.11 (1.06, 1.16)	1.03 (0.98, 1.07)
Q3	3838/838,579	1.18 (1.12, 1.23)	1.05 (1.00, 1.10)
Q4	3711/840,723	1.14 (1.08, 1.19)	0.98 (0.94, 1.03)
*P* for trend		<0.001	0.610
NO_2_, per 10-μg/m^3^ increase	--	1.15 (1.13, 1.17)	1.00 (0.98, 1.02)
Q1	3365/914,056	1.00 (Ref.)	1.00 (Ref.)
Q2	3688/879,294	1.14 (1.09, 1.19)	1.02 (0.98, 1.07)
Q3	3739/828,930	1.23 (1.17, 1.28)	1.02 (0.98, 1.07)
Q4	3918/804,805	1.32 (1.26, 1.39)	0.99 (0.94, 1.04)
*P* for trend		<0.001	0.695
NO_X_, per 20-μg/m^3^ increase	--	1.15 (1.13, 1.17)	1.02 (1.01, 1.05)
Q1	3254/911,938	1.00 (Ref.)	1.00 (Ref.)
Q2	3620/970,880	1.17 (1.11, 1.22)	1.06 (1.01, 1.12)
Q3	3634/819,160	1.24 (1.19, 1.30)	1.05 (1.00, 1.11)
Q4	4202/825,106	1.43 (1.36, 1.50)	1.07 (1.02, 1.13)
*P* for trend		<0.001	0.017

Model 1: Unadjusted

Model 2: Adjusted for age, gender, ethnicity, education level, employment status, household income, and Townsend deprivation index

*Abbreviations*: *MDD* major depressive disorder, *HR* hazards ratio, *CI* confidence interval, *PM*_*2.5*_ fine particulate matter with diameter ≤2.5μm, *PM*_*10*_ particulate matter with diameter ≤10μm, *NO*_*2*_ nitrogen dioxide, *NO*_*X*_ nitrogen oxides, *Ref* reference

*P* value for trend calculated treating the air pollution concentrations (quartile) as a continuous variable

**Fig. 2:**
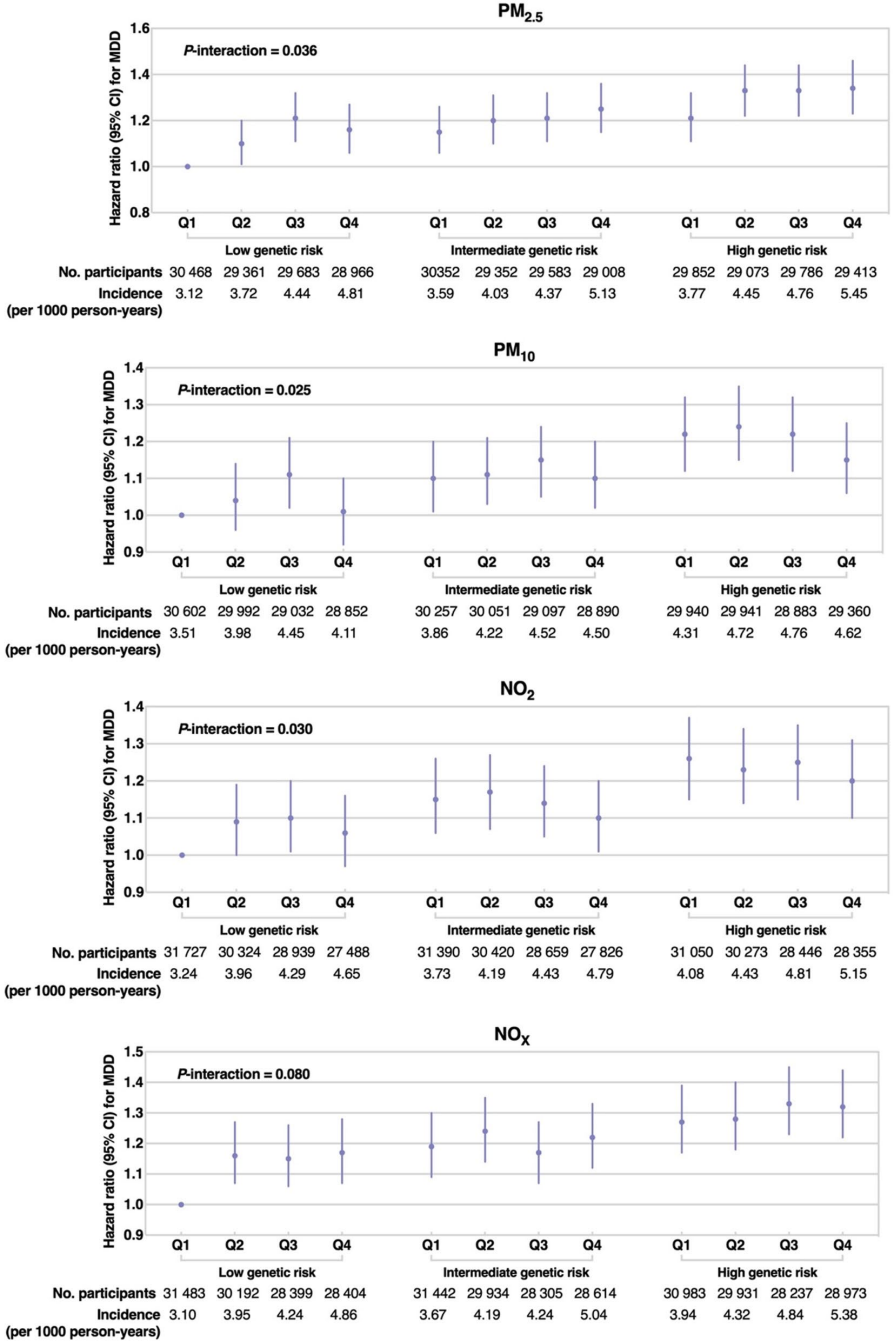
Joint associations of genetic risk score with incident major depressive disorder (MDD). Hazard ratios were adjusted for age, gender, ethnicity, education level, employment status, household income, and Townsend deprivation index. The interaction between genetic risk with air pollutants was tested by stratifying genetic risk

Additional file 1: Table S15 presents the associations of PRS with the risk of MDD. We found a significant association of PRS with MDD in the multivariable-adjusted model (HR: 1.10, 95% CI: 1.07–1.12). Figure 2 shows the risk of incident MDD for combined air pollutants and genetic risk. Compared with participants with low genetic risk and low air pollution exposure, those with high genetic risk and high PM_2.5_ exposures had the highest risk of incident MDD (PM_2.5_: HR: 1.34, 95% CI:1.23–1.46). The interaction effects between PRS with PM_2.5_, PM_10_, and NO_2_ on the risk of incident MDD were significant (*P*-interaction for PM_2.5_: 0.036; PM_10_: 0.025; NO_2_: 0.030; NO_x_: 0.080).

**Fig. 3:**
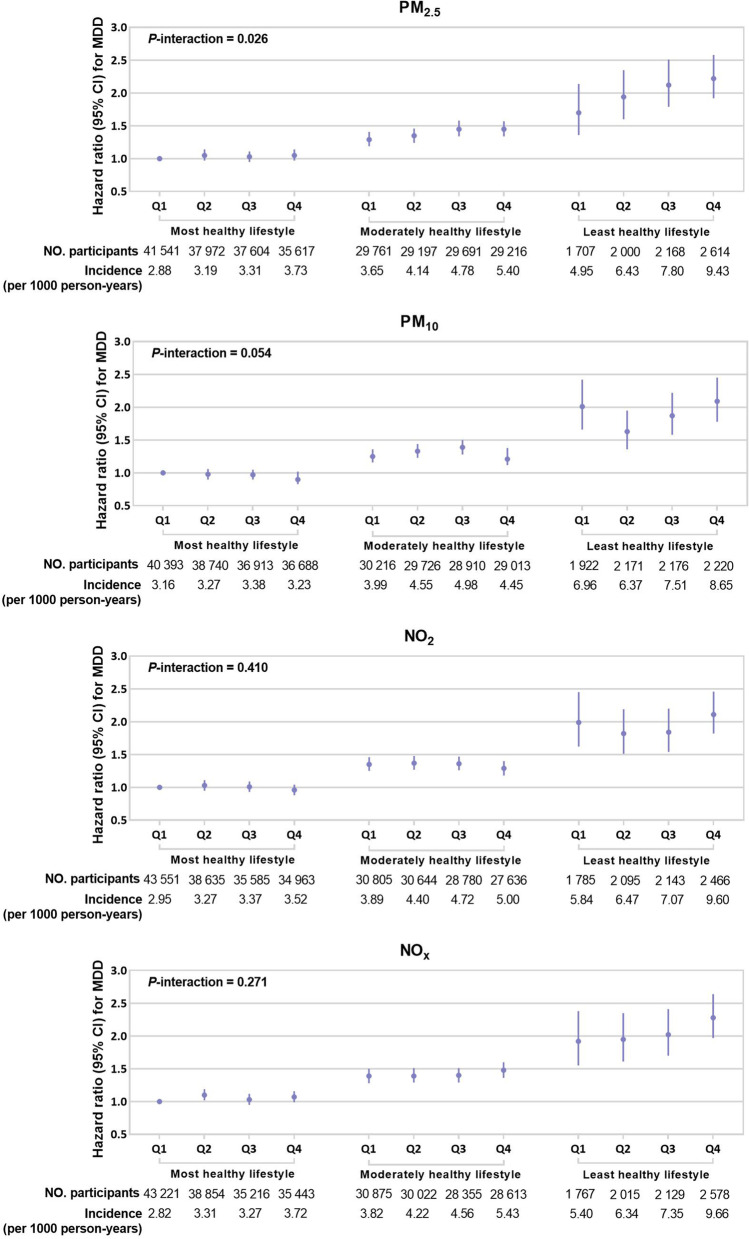
Joint associations of healthy lifestyle score with incident major depressive disorder (MDD). Hazard ratios were adjusted for age, gender, ethnicity, education level, employment status, household income, and Townsend deprivation index. The interaction between lifestyle with air pollutants was tested by stratifying lifestyle categories

The risks of incident MDD according to lifestyle categories and lifestyle score are provided in Additional file 1: Table S16-S17. MDD risk increased monotonically across lifestyle categories and scores. The risk of incident MDD was 98% higher among those who have the least healthy lifestyle compared with those who have the most healthy lifestyle (HR: 1.98, 95% CI: 1.81–2.16). Figure 3 shows the risk of incident MDD for combined air pollutants and lifestyle. The highest MDD risk was observed in participants with the least healthy lifestyle and high PM_2.5_, PM_10_, NO_2_, and NO_x_ exposures (PM_2.5_: HR: 2.22, 95% CI:1.92–2.58; PM_10_: HR: 2.09, 95% CI:1.78–2.45; NO_2_: HR: 2.11, 95% CI: 1.82–2.46; NO_x_: HR: 2.28, 95% CI: 1.97–2.64). The interaction effects between lifestyle with PM_2.5_ in relation to incident MDD risk were significant (*P*-interaction for PM_2.5_: 0.026; PM_10_: 0.054; NO_2_: 0.410; NO_x_: 0.271). The potential effect modifications of individual lifestyle factors on MDD risk are presented in Additional file 1: Table S18. We observed a significant interaction between PM_2.5_ and processed meat intake to MDD risk (*P*-interaction = 0.022).

## Discussion

In this large-scaled population-based longitudinal study, we found that long-term exposures to PM_2.5_ and NO_x_ were significantly associated with the incident MDD risk. The risk of incident MDD was higher among those with high genetic risk or unhealthy lifestyle compared with those with low genetic risk or healthy lifestyle. We also found significant interactions of genetic susceptibility and lifestyles with exposure to air pollution. Increased genetic susceptibility and unhealthy lifestyles may intensify the impact of long-term exposure to air pollution on the risk of MDD.

Research on the role of air pollution exposure in the development of MDD is limited. We comprehensively examined the association between long-term exposure to various air pollutants and the risk of incident MDD. We used air pollution data from 2010 to capture long-term air pollution exposure, consistent with previous research [12, 36–38]. Air pollution levels in England have been relatively stable over these years [39, 40]. As the fluctuation of the temporal trend of most air pollution was generally stable during the study period, the average values of air pollution could be used as a surrogate measure of long-term exposure [41]. The use of the average air pollution levels as the mean estimates in our sensitivity analysis further demonstrated the robustness of this approach. Our findings are consistent with those of previous studies. For example, in a prospective study of 27,270 participants in Korea, the researchers observed a 44% increase in the risk of MDD for each 10-μg/m^3^ increase in PM_2.5_ levels [42]. Similarly, a nationwide prospective cohort study in the USA found an association between exposure to PM_2.5_ and the onset of depression [32]. Meanwhile, there are some other studies that are not consistent with our findings. For example, Zhang et al. discovered that long-term exposure to outdoor PM_10_ was associated with the development of depression; nevertheless, they did not find this association for PM_2.5_ [43]. Notably, previous studies lacked large-scale cohort settings and primarily examined particulate matter exposure (e.g., PM_2.5_), excluding other gaseous pollutants (e.g., NO_x_), which led to inconsistent results regarding the role of long-term exposure to air pollution in the development of MDD. Our study provides new evidence for epidemiological studies on the association between air pollutants and MDD. Our findings may have implications for policy regulation and clinical trials because changes in policies and individual behavior may help reduce air pollution and thus help mitigate symptoms of MDD.

There are some underlying mechanisms that could shed light on the associations of air pollution with MDD. One of the underlying mechanisms is that oxidative stress and neuroinflammation pathways induced by air pollutants could stimulate the onset and progression of MDD [44]. Air pollutants can penetrate the lung tissue compartments, enter the circulatory system, and reach the brain, causing oxidative stress and inflammation of the central nervous system [45]. In addition, experimental and animal studies have observed that inhalation of particulate matter could stimulate increased expression of redox/glucocorticoid-sensitive genes in rats, suggesting the involvement of the hormonal pathway in mental health disorders associated with particulate matter [46]. However, determining which pathway offers the most critical link is difficult because of the existing scarcity of particle-specific translocation kinetics and exposure levels [47]. In addition, vascular disease is an essential intermediate factor in the association between air pollution exposure and an increased risk of subsequent MDD. Increasing bodies of evidence demonstrate that exposure to air pollution leads to cerebrovascular disease, which may affect the central nervous system and the brain, contributing to an increased risk of depression and other related conditions [48]. Previous studies have also indicated that vascular disease is associated with inflammatory pathway activation, leading to MDD or dysthymia [49]. Additional studies are necessary to determine the precise mechanisms underlying the air pollution–induced pathogenesis of MDD.

Previous research demonstrated that the etiology of MDD is multifactorial and that its heritability is approximately 35% [9]. Research also demonstrated that PRS may serve as an early indicator of clinically significant levels of depression and be associated with the risk of depression [50]. Our results are consistent with these findings. Additionally, we investigated the contribution of genetic susceptibility to the association between air pollution and MDD and found that air pollution may increase the risk of MDD, particularly among individuals with high genetic susceptibility. Li et al. explored how PM_2.5_ exposure interacts with polygenic risk in the development of MDD across multiple levels of brain network function [51]. They observed that a combination of exposure to high levels of air pollution and a relatively high polygenic risk for MDD disproportionately augmented stress-related effects on the brain circuitry. Working memory and stress-related information transfer across cortical and subcortical brain networks were influenced by PM_2.5_ exposures to differing extents depending on the polygenic risk for MDD in gene-by-environment interactions [51]. However, other explanations for these mechanisms can be applied when they are separated into particular variants. Previous studies have revealed that patients with psychiatric disorders had a higher mRNA expression level of vaccinia-related kinase 2 (VRK2) than did healthy individuals [52, 53]. In addition, a randomized crossover study suggested that higher PM_2.5_ exposure was positively associated with the mRNA expression of cytokine [54]. Therefore, air pollution may interact with rs1518395 located in *VRK2* to jointly affect the onset of MDD. In addition, some SNPs from an MDD GWAS, such as rs10514299, could be enriched in genes expressed in the central nervous system and function in transcriptional regulation related to neurodevelopment [10]. Because of their toxicity to the central nervous system, air pollutants may also contribute to the development of mental diseases [55]. Therefore, by affecting the central nervous system, SNPs and air pollution may contribute to the onset of depression. Accordingly, elucidating the pathophysiology of MDD is imperative.

In addition, we also confirmed that unhealthy lifestyles were associated with higher risks of MDD. Considering the complexity of health behaviors and that most health behaviors are interconnected, a comprehensive analysis of healthy lifestyles may better capture the impact of lifestyle than an analysis based on a single factor. Our findings are in concert with the previous studies. Adjibade and colleagues formulated a healthy lifestyle index that incorporates multiple lifestyle factors and discovered that combined healthy lifestyles were associated with a lower risk of depressive symptoms [56]. We also observed that the deleterious associations between PM_2.5_ and MDD were stronger among individuals who led unhealthy lifestyles. Indeed, besides long-term air pollution exposure may reach the brain through the lung–brain axis and induce systemic inflammation [57], unhealthy lifestyle factors have also been associated with elevated inflammation levels [58, 59]. Conversely, higher levels of systemic inflammation marker may contribute to the development of different neuropsychiatric disorders including depression [60]. Therefore, when air pollution and unhealthy lifestyle are employed together for MDD, it is reasonable to appear enhanced effect. These findings emphasize the importance of lifestyle changes. The benefit of air pollution exposure reduction in lowering the risk of MDD is expected to be greatest among individuals with healthy lifestyles; this finding can inform the establishment of personalized preventive strategies for reducing the risk of MDD.

To the best of our knowledge, our study is the first to evaluate the modifying effect of genetic susceptibility and lifestyles on the association between air pollution exposure and the risk of MDD. The main strengths of our study are its inclusion of a large sample size, prospective design, and consistent results in several sensitivity analyses. Nevertheless, we also acknowledge several limitations of our study. First, an exposure assessment based on a single address does not eliminate the possibility of exposure misclassification caused by outside activities. Further studies with more accurate estimates are needed to confirm the present findings. In addition, we had to admit that the effect of the collinearity cannot be ruled out, single-pollutant associations may be not independent, and the results should be interpreted with caution. Second, common to most previous environmental epidemiology studies [12, 36–38], we used the annual average air pollution concentration in 2010 as a proxy for the long-term air pollution exposure, which might induce the exposure misclassification. However, previous studies have suggested that the fluctuation of the temporal trend of most air pollution was generally stable during the study period in UK Biobank [12, 61, 62]. Furthermore, of note, similar results were found when a time-varying air pollution exposure was used in the sensitivity analysis, supporting the validity of using the baseline concentration. In addition, the UK Biobank lacks data on air pollution composition; therefore, there is still uncertainty as to which components are the most harmful. Third, incident MDD cases are not always well captured through hospital inpatient records and death registries. Although diagnosis by a doctor is a more common and precise way, some mildly depressed people may do not go to the hospital, resulting in MDD cases that were likely to be underreported. Fourth, a sample of 500,000 was recruited in UK Biobank with remarkable speed and efficiency, but this efficiency was achieved at the expense of a response rate (5.5%) and was subject to selection bias. Nevertheless, the absolute difference in these estimates was low and lead to the practical importance of such risk underestimation is likely to be small [63]. Fifth, additional MDD-related variants may be identified in future GWAS, the inclusion of additional SNPs in further study may help to further refine the estimation of genetic risk. Sixth, although we have adjusted for a series of potential confounders in our analysis, potential residual confounding from unmeasured or unknown variables might still be present. Finally, because the majority of our study’s participants were of European descent, the generalization of our findings regarding the associations of air pollution exposure and genetic susceptibility with MDD to other populations should be interpreted with caution.

## Conclusions

In summary, based on this large prospective cohort study, we found that long-term exposure to ambient air pollution was associated with a higher risk of MDD. High genetic risk and unhealthy lifestyle may intensify the impact of air pollution on MDD risk, highlighting the importance of identifying individuals with high genetic risk and developing healthy lifestyles for reducing the harm of air pollution to public mental health.

## Supplementary Information

**Additional file 1: Table S1.** Summary results of SNPs. **Table S2.** Variables used to create lifestyle score. **Table S3.** ICD-10 codes to assist in identifying MDD. **Table S4.** Summary statistics of air pollution data. **Table S5.** Sensitivity analysis by excluding MDD occurred in the first 2 years of follow-up. **Table S6.** Sensitivity analysis by excluding participants who live in the current address for less than 5 years. **Table S7.** Sensitivity analysis by excluding anxiety cases. **Table S8.** Sensitivity analysis by excluding dementia cases. **Table S9.** Sensitivity analysis after additional adjustment for other covariates. **Table S10.** Sensitivity analysis restricted to participants with complete covariates. **Table S11.** Sensitivity analysis was further linked primary care records. **Table S12.** Time-varying air pollution exposure and MDD. **Table S13.** Major principal components and MDD. **Table S14.** Stratified analysis by age and gender. **Table S15.** Genetic risk and MDD. **Table S16.** Lifestyle category and MDD. **Table S17**. MDD risk according to lifestyle score. **Table S18.** Stratified analysis by lifestyle factors. **Figure S1.** Flow chat. **Figure S2.** The description of time line. **Figure S3.** Directed Acyclic Graph. **Figure S4.** Schoenfeld residuals test for PM_2.5_. **Figure S5.** Schoenfeld residuals test for PM_10_. **Figure S6.** Schoenfeld residuals test for NO_2_. **Figure S7.** Schoenfeld residuals test for NO_x_. **Figure S8.** Pearson correlations between air pollution. **Figure S9.** Distribution of MDD genetic risk score.

## Data Availability

The data used in this current study are available from the UK Biobank data resources. Permissions are required in order to gain access to the UK Biobank data resources, subject to successful registration and application process. Further information can be found on the UK Biobank website (https://www.ukbiobank.ac.uk/).

## Abbreviations

CI: Confidence interval
DAG: Directed acyclic graph
FCS: Fully conditional specification
GWAS: Genome-wide association study
HR: Hazards ratio
ICD-10: International Classification of Diseases, Tenth Revision
LUR: Land Use Regression
MDD: Major depressive disorder
NO_2_: Nitrogen dioxide
NO_x_: Nitrogen oxides
PCA: Principal component analysis
PM_10_: Particulate matter with aerodynamic diameter ≤ 10μm
PM_2.5_: Particulate matter with aerodynamic diameter ≤ 2.5μm
PRSs: Polygenic risk scores
SD: Standard deviation
SNPs: Single-nucleotide polymorphisms
VRK2: Vaccinia-related kinase 2
