# Supplementary material for: Genetic susceptibility and lifestyle modify the association of long-term air pollution exposure on major depressive disorder: a prospective study in UK Biobank
Source: BMC Med. 2023 Feb 21;21:67. doi: 10.1186/s12916-023-02783-0 (PMC9945634; doi:10.1186/s12916-023-02783-0)
Supplement: Supplementary file 1 — Additional file 1: Table S1. Summary results of SNPs. Table S2. Variables used to create lifestyle score. Table S3. ICD-10 codes to assist in identifying MDD. Table S4. Summary statistics of air pollution data. Table S5. Sensitivity analysis by excluding MDD occurred in the first 2 years of follow-up. Table S6. Sensitivity analysis by excluding participants who live in the current address for less than 5 years. Table S7. Sensitivity analysis by excluding anxiety cases. Table S8. Sensitivity analysis by excluding dementia cases. Table S9. Sensitivity analysis after additional adjustment for other covariates. Table S10. Sensitivity analysis restricted to participants with complete covariates. Table S11. Sensitivity analysis was further linked primary care records. Table S12. Time-varying air pollution exposure and MDD. Table S13. Major principal components and MDD. Table S14. Stratified analysis by age and gender. Table S15. Genetic risk and MDD. Table S16. Lifestyle category and MDD. Table S17. MDD risk according to lifestyle score. Table S18. Stratified analysis by lifestyle factors. Figure S1. Flow chat. Figure S2. The description of time line. Figure S3. Directed Acyclic Graph. Figure S4. Schoenfeld residuals test for PM2.5. Figure S5. Schoenfeld residuals test for PM10. Figure S6. Schoenfeld residuals test for NO2. Figure S7. Schoenfeld residuals test for NOx. Figure S8. Pearson correlations between air pollution. Figure S9. Distribution of MDD genetic risk score. [file 12916_2023_2783_MOESM1_ESM.docx]

**Additional file 1**

**Genetic susceptibility and lifestyle modify the association of long-term air pollution exposure on major depressive disorder: a prospective study in UK Biobank**

Dankang Li, Junqing Xie, Lulin Wang, Yu Sun, Yonghua Hu, and Yaohua Tian

**Table S1**. Summary results of 17 SNPs used for polygenic risk score.

| No. | SNP ID | Chromosome | Position (bp) | Gene Context | Alleles | EAF | Beta |
| --- | --- | --- | --- | --- | --- | --- | --- |
| 1 | rs11209948 | 1 | 72811904 | NEGR1--[] | G/T | 0.6389 | 0.0357 |
| 2 | rs301806 | 1 | 8482078 | [RERE] | C/T | 0.5519 | -0.0271 |
| 3 | rs12065553 | 1 | 80793118 | [] | A/G | 0.2797 | 0.0309 |
| 4 | rs2422321 | 1 | 73293393 | NEGR1---[] | A/G | 0.4410 | 0.0308 |
| 5 | rs1518395 | 2 | 58208074 | []--VRK2 | A/G | 0.6130 | 0.0320 |
| 6 | rs1656369 | 3 | 158280085 | RSRC1--[]-MLF1 | A/T | 0.6703 | 0.0346 |
| 7 | rs10514299 | 5 | 87663610 | TMEM161B--[]---MEF2C | C/T | 0.2406 | 0.0449 |
| 8 | rs454214 | 5 | 88003403 | TMEM161B---[]--MEF2C | C/T | 0.5681 | -0.0337 |
| 9 | rs4543289 | 5 | 164484948 | [] | G/T | 0.4801 | -0.0322 |
| 10 | rs1475120 | 6 | 105389953 | HACE1--[]--LIN28B | A/G | 0.4525 | -0.0292 |
| 11 | rs6476606 | 9 | 37005561 | [PAX5] | A/G | 0.6372 | -0.0277 |
| 12 | rs7044150 | 9 | 2982931 | KIAA0020---[]---RFX3 | C/T | 0.3845 | -0.0299 |
| 13 | rs10786831 | 10 | 106614571 | [SORCS3] | A/G | 0.6007 | -0.0297 |
| 14 | rs2125716 | 12 | 84941429 | []---SLC6A15 | A/G | 0.7655 | -0.0345 |
| 15 | rs12552 | 13 | 53625781 | [OLFM4] | A/G | 0.5550 | -0.0433 |
| 16 | rs8025231 | 15 | 37648402 | MEIS2---[]---TMCO5A | A/C | 0.4275 | 0.0355 |
| 17 | rs2179744 | 22 | 41621714 | [L3MBTL2] | A/G | 0.7183 | -0.0354 |

Abbreviations: SNP, single nucleotide polymorphism; EAF, effect allele frequency.

**Table S2.** Variables used to create lifestyle score for UK Biobank.

| **Lifestyle factor** | **Questionnaire** | **‘Healthy’** | **‘Unhealthy’** |
| --- | --- | --- | --- |
| Smoking status | "Do you smoke tobacco now?" and "In the past, how often have you smoked tobacco?" | Past or never smoker | Current |
| Alcohol intake | “About how often do you drink alcohol?” | ≤ 4 times week | Daily or almost daily |
| Physical activity | IPAQ short form2– total time walking or moderate and vigorous-intensity physical activity (PA) in previous week | ≥150 min/week moderate or ≥ 75 min/week vigorous PA | <150 min/week moderate or < 75 min/week vigorous PA |
| TV viewing | "In a typical day, how many hours do you spend watching TV?" | < 4 h/day | ≥ 4 h/day |
| Sleep time | “About how many hours sleep do you get in every 24 hours?” | >7 or <9h/day | <7 or >9h/day |
| Dietary characteristics | Individual dietary components contributed directly to lifestyle score. |  |  |
| Fruit and vegetable intake | "About how many of …. would you eat per day?” Separate questions for pieces of fresh and dried fruit, tablespoons of salad or cooked/raw vegetables. Combined and converted to g/day (1 portion = 80 g) | ≥ 400 g/day | <400 g/ day |
| Oily fish intake | "How often do you eat oily fish? (e.g. sardines, salmon, mackerel, herring)" | ≥1 portion/week | <1 portion/week |
| Red meat intake | "How often do you eat…?” Separate questions for Beef / lamb or mutton / pork (excluding processed meats such as ham or bacon). Red meat included due to clear link between red meat and mortality. | ≤3 portion/week | >3 portion/week |
| Processed meat intake | "How often do you eat processed meats (such as bacon, ham, sausages, meat pies, kebabs, burgers, chicken nuggets)?" | ≤1 portion/week | >1 portion/week |

Participants were classified to healthy or unhealthy categories based on their responses to questions for each lifestyle factor. UK Biobank physical activity (PA) data were analyzed in accordance with the International Physical Activity Questionnaire (IPAQ) scoring protocol with total physical activity computed as the sum of walking, moderate and vigorous activity, measured as metabolic equivalents (MET-hours/week). National dietary guidelines were used as the basis for the dietary components. UK Biobank dietary information was collected via the Oxford WebQ; a web-based 24-hour recall questionnaire developed specifically for use in large population studies.

**Table S3.** International Classification of Diseases (ICD) - 10 codes used to identify major depressive disorder (MDD) diagnosis.

| Major depressive disorder (MDD) | ICD-10 |
| --- | --- |
| Depressive episode | F32 |
| Mild depressive episode | F32.0 |
| Moderate depressive episode | F32.1 |
| Severe depressive episode without psychotic symptoms | F32.2 |
| Severe depressive episode with psychotic symptoms | F32.3 |
| Other depressive episodes | F32.8 |
| Depressive episode, unspecified | F32.9 |
| Recurrent depressive disorder | F33 |
| Recurrent depressive disorder, current episode mild | F33.0 |
| Recurrent depressive disorder, current episode moderate | F33.1 |
| Recurrent depressive disorder, current episode severe without psychotic symptoms | F33.2 |
| Recurrent depressive disorder, current episode severe with psychotic symptoms | F33.3 |
| Recurrent depressive disorder, currently in remission | F33.4 |
| Other recurrent depressive disorders | F33.8 |
| Recurrent depressive disorder, unspecified | F33.9 |

Abbreviations: ICD, International Classification of Diseases.

**Table S4.** Summary statistics of air pollution data.

| Air pollution, μg/m^3^ | Mean ± SD | Percentiles | | | | |
| --- | --- | --- | --- | --- | --- | --- |
|  |  | Minimum | 25th | 50th | 75th | Maximum |
| PM_2.5_ | 9.98 ± 1.06 | 8.17 | 9.27 | 9.92 | 10.55 | 21.31 |
| PM_10_ | 16.20 ± 1.90 | 11.78 | 15.21 | 16.00 | 16.98 | 30.65 |
| NO_2_ | 26.41 ± 7.59 | 12.93 | 21.16 | 25.84 | 30.90 | 108.31 |
| NO_X_ | 43.68 ± 15.55 | 19.74 | 33.85 | 41.88 | 50.43 | 265.94 |

Abbreviation: SD, standard deviation; PM_2.5_, fine particulate matter with diameter ≤2.5μm; PM_10_, particulate matter with diameter ≤10μm; NO_2_, nitrogen dioxide; NO_X_, nitrogen oxides.

**Table S5.** Association between long-term exposure to air pollution and major depressive disorder (MDD) by excluding MDD cases occurred in the first 2 years of follow-up.

| Air pollution | HR (95%CI) for Continuous* | Air pollution concentrations (quartile) | | | | P for trend |
| --- | --- | --- | --- | --- | --- | --- |
|  |  | Q1 | Q2 | Q3 | Q4 |  |
| PM_2.5_ |  |  |  |  |  |  |
| Model 1 | 1.85 (1.71, 2.00) | 1.00 (Ref.) | 1.16 (1.11, 1.23) | 1.28 (1.22, 1.35) | 1.44 (1.37, 1.51) | <0.001 |
| Model 2 | 1.13 (1.03, 1.24) | 1.00 (Ref.) | 1.08 (1.03, 1.14) | 1.10 (1.05, 1.16) | 1.10 (1.04, 1.16) | 0.001 |
| PM_10_ |  |  |  |  |  |  |
| Model 1 | 1.28 (1.17, 1.40) | 1.00 (Ref.) | 1.10 (1.05, 1.16) | 1.17 (1.11, 1.23) | 1.13 (1.07, 1.19) | <0.001 |
| Model 2 | 1.00 (0.91, 1.10) | 1.00 (Ref.) | 1.02 (0.97, 1.07) | 1.04 (0.99, 1.10) | 0.98 (0.93, 1.03) | 0.607 |
| NO_2_ |  |  |  |  |  |  |
| Model 1 | 1.14 (1.12, 1.17) | 1.00 (Ref.) | 1.12 (1.06, 1.18) | 1.21 (1.15, 1.27) | 1.30 (1.23, 1.36) | <0.001 |
| Model 2 | 1.00 (0.97, 1.02) | 1.00 (Ref.) | 1.01 (0.96, 1.06) | 1.01 (0.96, 1.07) | 0.98 (0.92, 1.03) | 0.523 |
| NO_X_ |  |  |  |  |  |  |
| Model 1 | 1.14 (1.12, 1.17) | 1.00 (Ref.) | 1.15 (1.10, 1.22) | 1.23 (1.17, 1.29) | 1.41 (1.34, 1.48) | <0.001 |
| Model 2 | 1.02 (1.00, 1.04) | 1.00 (Ref.) | 1.06 (1.00, 1.11) | 1.04 (0.99, 1.10) | 1.07 (1.01, 1.13) | 0.049 |

Abbreviations: MDD, major depressive disorder; HR, hazards ratio; CI, confidence interval; PM_2.5_, fine particulate matter with diameter ≤2.5μm; PM_10_, particulate matter with diameter ≤10μm; NO_2_, nitrogen dioxide; NO_X_, nitrogen oxides; Ref, reference.

*Hazards ratio (HR) per 5-μg/m^3^ change of PM_2.5_ level, per 10-μg/m^3^ change of PM_10_ and NO_2_ levels and per 20-μg/m^3^ change of NO_X_ level.

P value for trend calculated treating the air pollution concentrations (quartile) as a continuous variable.

Model 1: Unadjusted.

Model 2: Adjusted for age, gender, ethnicity, education level, employment status, household income, and Townsend deprivation index.

**Table S6.** Association between long-term exposure to air pollution and major depressive disorder (MDD) by excluding participants who live in the current address for less than 5 years.

| Air pollution | HR (95%CI) for Continuous* | Air pollution concentrations (quartile) | | | | P for trend |
| --- | --- | --- | --- | --- | --- | --- |
|  |  | Q1 | Q2 | Q3 | Q4 |  |
| PM_2.5_ |  |  |  |  |  |  |
| Model 1 | 1.86 (1.72, 2.02) | 1.00 (Ref.) | 1.16 (1.10, 1.22) | 1.28 (1.21, 1.34) | 1.44 (1.37, 1.51) | <0.001 |
| Model 2 | 1.14 (1.04, 1.25) | 1.00 (Ref.) | 1.08 (1.02, 1.14) | 1.10 (1.05, 1.16) | 1.10 (1.04, 1.16) | 0.001 |
| PM_10_ |  |  |  |  |  |  |
| Model 1 | 1.24 (1.13, 1.36) | 1.00 (Ref.) | 1.09 (1.03, 1.14) | 1.17 (1.11, 1.23) | 1.11 (1.05, 1.17) | <0.001 |
| Model 2 | 0.96 (0.87, 1.06) | 1.00 (Ref.) | 1.02 (0.96, 1.07) | 1.05 (1.00, 1.10) | 0.96 (0.91, 1.02) | 0.316 |
| NO_2_ |  |  |  |  |  |  |
| Model 1 | 1.14 (1.11, 1.17) | 1.00 (Ref.) | 1.11 (1.06, 1.17) | 1.20 (1.14, 1.26) | 1.28 (1.22, 1.35) | <0.001 |
| Model 2 | 0.99 (0.96, 1.02) | 1.00 (Ref.) | 1.01 (0.96, 1.06) | 1.01 (0.96, 1.06) | 0.96 (0.91, 1.02) | 0.232 |
| NO_X_ |  |  |  |  |  |  |
| Model 1 | 1.14 (1.12, 1.16) | 1.00 (Ref.) | 1.14 (1.08, 1.20) | 1.21 (1.15, 1.28) | 1.38 (1.31, 1.45) | <0.001 |
| Model 2 | 1.01 (1.00, 1.11) | 1.00 (Ref.) | 1.05 (1.00, 1.11) | 1.03 (0.98, 1.09) | 1.04 (0.99, 1.11) | 0.218 |

Abbreviations: MDD, major depressive disorder; HR, hazards ratio; CI, confidence interval; PM_2.5_, fine particulate matter with diameter ≤2.5μm; PM_10_, particulate matter with diameter ≤10μm; NO_2_, nitrogen dioxide; NO_X_, nitrogen oxides; Ref, reference.

*Hazards ratio (HR) per 5-μg/m^3^ change of PM_2.5_ level, per 10-μg/m^3^ change of PM_10_ and NO_2_ levels and per 20-μg/m^3^ change of NO_X_ level.

P value for trend calculated treating the air pollution concentrations (quartile) as a continuous variable.

Model 1: Unadjusted.

Model 2: Adjusted for age, gender, ethnicity, education level, employment status, household income, and Townsend deprivation index.

**Table S7.** Association between long-term exposure to air pollution and major depressive disorder (MDD) by excluding anxiety cases during follow-up.

| Air pollution | HR (95%CI) for Continuous* | Air pollution concentrations (quartile) | | | | P for trend |
| --- | --- | --- | --- | --- | --- | --- |
|  |  | Q1 | Q2 | Q3 | Q4 |  |
| PM_2.5_ |  |  |  |  |  |  |
| Model 1 | 1.88 (1.73, 2.06) | 1.00 (Ref.) | 1.15 (1.08, 1.22) | 1.28 (1.21, 1.36) | 1.44 (1.36, 1.52) | <0.001 |
| Model 2 | 1.14 (1.03, 1.26) | 1.00 (Ref.) | 1.07 (1.00, 1.13) | 1.10 (1.04, 1.17) | 1.09 (1.03, 1.16) | 0.004 |
| PM_10_ |  |  |  |  |  |  |
| Model 1 | 1.28 (1.16, 1.42) | 1.00 (Ref.) | 1.10 (1.04, 1.17) | 1.16 (1.09, 1.22) | 1.12 (1.06, 1.19) | <0.001 |
| Model 2 | 0.98 (0.88, 1.09) | 1.00 (Ref.) | 1.02 (0.97, 1.08) | 1.03 (0.97, 1.09) | 0.97 (0.91, 1.03) | 0.281 |
| NO_2_ |  |  |  |  |  |  |
| Model 1 | 1.14 (1.11, 1.17) | 1.00 (Ref.) | 1.10 (1.04, 1.16) | 1.18 (1.12, 1.25) | 1.26 (1.19, 1.33) | <0.001 |
| Model 2 | 0.98 (0.96, 1.01) | 1.00 (Ref.) | 0.99 (0.94, 1.05) | 0.98 (0.93, 1.04) | 0.93 (0.87, 1.00) | 0.033 |
| NO_X_ |  |  |  |  |  |  |
| Model 1 | 1.15 (1.12, 1.17) | 1.00 (Ref.) | 1.14 (1.08, 1.21) | 1.22 (1.15, 1.29) | 1.39 (1.32, 1.47) | <0.001 |
| Model 2 | 1.02 (0.99, 1.05) | 1.00 (Ref.) | 1.05 (0.99, 1.11) | 1.03 (0.97, 1.09) | 1.04 (0.98, 1.11) | 0.309 |

Abbreviations: MDD, major depressive disorder; HR, hazards ratio; CI, confidence interval; PM_2.5_, fine particulate matter with diameter ≤2.5μm; PM_10_, particulate matter with diameter ≤10μm; NO_2_, nitrogen dioxide; NO_X_, nitrogen oxides; Ref, reference.

*Hazards ratio (HR) per 5-μg/m^3^ change of PM_2.5_ level, per 10-μg/m^3^ change of PM_10_ and NO_2_ levels and per 20-μg/m^3^ change of NO_X_ level.

P value for trend calculated treating the air pollution concentrations (quartile) as a continuous variable.

Model 1: Unadjusted.

Model 2: Adjusted for age, gender, ethnicity, education level, employment status, household income, and Townsend deprivation index.

**Table S8.** Association between long-term exposure to air pollution and major depressive disorder (MDD) by excluding dementia cases during follow-up.

| Air pollution | HR (95%CI) for Continuous* | Air pollution concentrations (quartile) | | | | P for trend |
| --- | --- | --- | --- | --- | --- | --- |
|  |  | Q1 | Q2 | Q3 | Q4 |  |
| PM_2.5_ |  |  |  |  |  |  |
| Model 1 | 1.92 (1.78, 2.06) | 1.00 (Ref.) | 1.16 (1.11, 1.22) | 1.30 (1.23, 1.36) | 1.47 (1.40, 1.55) | <0.001 |
| Model 2 | 1.14 (1.05, 1.24) | 1.00 (Ref.) | 1.07 (1.02, 1.13) | 1.11 (1.05, 1.16) | 1.11 (1.05, 1.16) | <0.001 |
| PM_10_ |  |  |  |  |  |  |
| Model 1 | 1.29 (1.19, 1.41) | 1.00 (Ref.) | 1.10 (1.05, 1.15) | 1.18 (1.13, 1.24) | 1.13 (1.08, 1.19) | <0.001 |
| Model 2 | 0.98 (0.90, 1.08) | 1.00 (Ref.) | 1.02 (0.98, 1.08) | 1.04 (1.00, 1.11) | 0.97 (0.93, 1.02) | 0.471 |
| NO_2_ |  |  |  |  |  |  |
| Model 1 | 1.15 (1.12, 1.17) | 1.00 (Ref.) | 1.14 (1.08, 1.19) | 1.22 (1.16, 1.28) | 1.32 (1.26, 1.38) | <0.001 |
| Model 2 | 0.99 (0.96, 1.02) | 1.00 (Ref.) | 1.02 (0.97, 1.07) | 1.01 (0.96, 1.06) | 0.97 (0.92, 1.03) | 0.306 |
| NO_X_ |  |  |  |  |  |  |
| Model 1 | 1.15 (1.13, 1.17) | 1.00 (Ref.) | 1.16 (1.11, 1.22) | 1.25 (1.19, 1.31) | 1.43 (1.37, 1.50) | <0.001 |
| Model 2 | 1.02 (1.00, 1.04) | 1.00 (Ref.) | 1.06 (1.01, 1.11) | 1.05 (1.00, 1.10) | 1.07 (1.01, 1.12) | 0.032 |

Abbreviations: MDD, major depressive disorder; HR, hazards ratio; CI, confidence interval; PM_2.5_, fine particulate matter with diameter ≤2.5μm; PM_10_, particulate matter with diameter ≤10μm; NO_2_, nitrogen dioxide; NO_X_, nitrogen oxides; Ref, reference.

*Hazards ratio (HR) per 5-μg/m^3^ change of PM_2.5_ level, per 10-μg/m^3^ change of PM_10_ and NO_2_ levels and per 20-μg/m^3^ change of NO_X_ level.

P value for trend calculated treating the air pollution concentrations (quartile) as a continuous variable.

Model 1: Unadjusted.

Model 2: Adjusted for age, gender, ethnicity, education level, employment status, household income, and Townsend deprivation index.

**Table S9.** Association between long-term exposure to air pollution and major depressive disorder (MDD) after additional adjustment for other covariates.

| Air pollution | HR (95%CI) for Continuous* | Air pollution concentrations (quartile) | | | | *P* for trend |
| --- | --- | --- | --- | --- | --- | --- |
|  |  | Q1 | Q2 | Q3 | Q4 |  |
| PM_2.5_ | 1.16 (1.05, 1.28) | 1.00 (Ref.) | 1.04 (0.98, 1.11) | 1.07 (1.01 1.14) | 1.10 (1.04, 1.17) | <0.001 |
| PM_10_ | 0.93 (0.84, 1.04) | 1.00 (Ref.) | 1.00 (0.95, 1.06) | 1.03 (0.98, 1.09) | 0.93 (0.88, 1.00) | 0.051 |
| NO_2_ | 1.00 (0.97, 1.03) | 1.00 (Ref.) | 1.01 (0.96, 1.07) | 1.00 (0.94, 1.06) | 0.97 (0.91, 1.04) | 0.384 |
| NO_X_ | 1.02 (1.00, 1.05) | 1.00 (Ref.) | 1.05 (1.00, 1.11) | 1.02 (0.96, 1.08) | 1.08 (1.02, 1.15) | 0.041 |

Abbreviations: MDD, major depressive disorder; HR, hazards ratio; CI, confidence interval; PM_2.5_, fine particulate matter with diameter ≤2.5μm; PM_10_, particulate matter with diameter ≤10μm; NO_2_, nitrogen dioxide; NO_X_, nitrogen oxides; Ref, reference.

*Hazards ratio (HR) per 5-μg/m^3^ change of PM_2.5_ level, per 10-μg/m^3^ change of PM_10_ and NO_2_ levels and per 20-μg/m^3^ change of NO_X_ level.

*P* value for trend calculated treating the air pollution concentrations (quartile) as a continuous variable.

Multifactorial adjustments were made for age, gender, ethnicity, education level, employment status, household income, Townsend deprivation index, BMI categories (< 25 kg/m² as normal weight; ≥ 25 kg/m² and < 30 kg/m² as overweight; or ≥ 30 kg/m² as obesity), cardiometabolic disease (none; high blood pressure; heart attack, angina, or stroke; or both high blood pressure and heart attack, angina, or stroke), diabetes (none or yes), and lifestyle categories (more healthy, moderately healthy, and least healthy), and MDD-PRS.

**Table S10.** Association between long-term exposure to air pollution and major depressive disorder (MDD) restricted to participants with complete covariates.

| Air pollution | HR (95%CI) for Continuous* | Air pollution concentrations (quartile) | | | | *P* for trend |
| --- | --- | --- | --- | --- | --- | --- |
|  |  | Q1 | Q2 | Q3 | Q4 |  |
| PM_2.5_ | 1.18 (1.08, 1.30) | 1.00 (Ref.) | 1.08 (1.02, 1.14) | 1.10 (1.05, 1.16) | 1.13 (1.07, 1.19) | <0.001 |
| PM_10_ | 1.02 (0.92, 1.12) | 1.00 (Ref.) | 1.03 (0.98, 1.08) | 1.05 (0.99, 1.10) | 0.99 (0.93, 1.04) | 0.720 |
| NO_2_ | 1.01 (0.98, 1.03) | 1.00 (Ref.) | 1.04 (1.00, 1.10) | 1.04 (0.98, 1.09) | 1.00 (0.95, 1.06) | 0.971 |
| NO_X_ | 1.03 (1.00, 1.05) | 1.00 (Ref.) | 1.05 (1.00, 1.11) | 1.04 (1.00, 1.10) | 1.07 (1.01, 1.13) | 0.030 |

Abbreviations: MDD, major depressive disorder; HR, hazards ratio; CI, confidence interval; PM_2.5_, fine particulate matter with diameter ≤2.5μm; PM_10_, particulate matter with diameter ≤10μm; NO_2_, nitrogen dioxide; NO_X_, nitrogen oxides; Ref, reference.

*Hazards ratio (HR) per 5-μg/m^3^ change of PM_2.5_ level, per 10-μg/m^3^ change of PM_10_ and NO_2_ levels and per 20-μg/m^3^ change of NO_X_ level.

*P* value for trend calculated treating the air pollution concentrations (quartile) as a continuous variable.

Multifactorial adjustments were made for age, gender, ethnicity, education level, employment status, household income, and Townsend deprivation index.

**Table S11.** Association between long-term exposure to air pollution and major depressive disorder (MDD) was derived from linkage to both hospital inpatient and primary care records.

| Air pollution | HR (95%CI) for Continuous* | Air pollution concentrations (quartile) | | | | *P* for trend |
| --- | --- | --- | --- | --- | --- | --- |
|  |  | Q1 | Q2 | Q3 | Q4 |  |
| PM_2.5_ | 1.21 (1.12, 1.31) | 1.00 (Ref.) | 1.09 (1.03, 1.15) | 1.12 (1.06, 1.18) | 1.15 (1.09, 1.21) | <0.001 |
| PM_10_ | 1.04 (0.94, 1.15) | 1.00 (Ref.) | 1.06 (1.00, 1.11) | 1.07 (1.02, 1.13) | 1.00 (0.94, 1.06) | 0.894 |
| NO_2_ | 1.02 (1.00, 1.05) | 1.00 (Ref.) | 1.04 (0.98, 1.10) | 1.06 (1.00, 1.12) | 1.04 (0.98, 1.10) | 0.200 |
| NO_X_ | 1.03 (1.01, 1.06) | 1.00 (Ref.) | 1.07 (1.01, 1.13) | 1.05 (0.99, 1.11) | 1.09 (1.03, 1.15) | 0.017 |

Abbreviations: MDD, major depressive disorder; HR, hazards ratio; CI, confidence interval; DAG, directed acyclic graph; PM_2.5_, fine particulate matter with diameter ≤2.5μm; PM_10_, particulate matter with diameter ≤10μm; NO_2_, nitrogen dioxide; NO_X_, nitrogen oxides; Ref, reference.

*Hazards ratio (HR) per 5-μg/m^3^ change of PM_2.5_ level, per 10-μg/m^3^ change of PM_10_ and NO_2_ levels and per 20-μg/m^3^ change of NO_X_ level.

*P* value for trend calculated treating the air pollution concentrations (quartile) as a continuous variable.

Multifactorial adjustments were made for age, gender, ethnicity, education level, employment status, household income, and Townsend deprivation index.

**Table S12.** Association between time-varying exposure to air pollution and major depressive disorder (MDD).

| Air pollution | HR (95%CI) for Continuous* | Air pollution concentrations (quartile) | | | | *P* for trend |
| --- | --- | --- | --- | --- | --- | --- |
|  |  | Q1 | Q2 | Q3 | Q4 |  |
| PM_2.5_ | 1.81 (1.73, 1.89) | 1.00 (Ref.) | 1.81 (1.72, 1.91) | 1.84 (1.74, 1.93) | 1.81 (1.72, 1.91) | <0.001 |
| PM_10_ | 1.20 (1.13, 1.27) | 1.00 (Ref.) | 1.14 (1.09, 1.20) | 1.23 (1.17, 1.29) | 1.12 (1.07, 1.18) | <0.001 |
| NO_2_ | 1.13 (1.11, 1.15) | 1.00 (Ref.) | 1.60 (1.52, 1.70) | 1.90 (1.80, 2.00) | 1.59 (1.51, 1.68) | <0.001 |
| NO_x_ | 1.11 (1.09, 1.12) | 1.00 (Ref.) | 1.58 (1.50, 1.65) | 1.83 (1.74, 1.92) | 1.54 (1.46, 1.62) | <0.001 |

Abbreviations: MDD, major depressive disorder; HR, hazards ratio; CI, confidence interval; DAG, directed acyclic graph; PM_2.5_, fine particulate matter with diameter ≤2.5μm; PM_10_, particulate matter with diameter ≤10μm; NO_2_, nitrogen dioxide; NO_X_, nitrogen oxides; Ref, reference.

*Hazards ratio (HR) per 5-μg/m^3^ change of PM_2.5_ level, per 10-μg/m^3^ change of PM_10_ and NO_2_ levels and per 20-μg/m^3^ change of NO_X_ level.

*P* value for trend calculated treating the air pollution concentrations (quartile) as a continuous variable.

Multifactorial adjustments were made for age, gender, ethnicity, education level, employment status, household income, and Townsend deprivation index.

**Table S13.** Associations between the three major principal components and MDD risk.

| Principal component | HR (95%CI) for Continuous | Air pollution score (quartile) | | | | *P* for trend |
| --- | --- | --- | --- | --- | --- | --- |
|  |  | Q1 | Q2 | Q3 | Q4 |  |
| PC1 | 1.02 (1.01, 1.04) | 1.00 (Ref.) | 1.06 (1.01, 1.11) | 1.07 (1.02, 1.12) | 1.08 (1.02, 1.13) | <0.001 |
| PC2 | 1.00 (0.98, 1.01) | 1.00 (Ref.) | 0.98 (0.94, 1.03) | 0.99 (0.94, 1.03) | 0.97 (0.93, 1.02) | 0.669 |

Abbreviations: MDD, major depressive disorder; HR, hazards ratio; CI, confidence interval; Ref, reference.

*P* value for trend calculated treating the air pollution score (quartile) as a continuous variable.

Multifactorial adjustments were made for age, gender, ethnicity, education level, employment status, household income, and Townsend deprivation index.

**Table S14**. Association between long-term exposure to air pollution and major depressive disorder (MDD) in subgroups stratified by age and gender.

| Air pollutions | <60 | |  | ≥60 | | P-value for interaction |  | Women | |  | Man | | *P*-value for interaction |
| --- | --- | --- | --- | --- | --- | --- | --- | --- | --- | --- | --- | --- | --- |
|  | HR (95% CI) | P |  | HR (95% CI) | P |  |  | HR (95% CI) | P |  | HR (95% CI) | P |  |
| PM_2.5_ | 1.11 (1.00, 1.23) | 0.056 |  | 1.28 (1.12, 1.46) | <0.001 | 0.572 |  | 1.11 (1.00, 1.23) | 0.058 |  | 1.24 (1.09, 1.42) | 0.001 | 0.049 |
| PM_10_ | 1.02 (0.91, 1.14) | 0.708 |  | 0.98 (0.85, 1.13) | 0.787 | 0.467 |  | 0.98 (0.88, 1.10) | 0.755 |  | 1.03 (0.89, 1.19) | 0.712 | 0.428 |
| NO_2_ | 0.98 (0.95, 1.01) | 0.232 |  | 1.03 (0.99, 1.07) | 0.104 | 0.243 |  | 0.99 (0.96, 1.02) | 0.473 |  | 1.01 (0.98, 1.06) | 0.467 | 0.077 |
| NO_X_ | 1.01 (0.98, 1.04) | 0.393 |  | 1.05 (1.01, 1.08) | 0.011 | 0.538 |  | 1.02 (1.00, 1.05) | 0.103 |  | 1.02 (0.99, 1.06) | <0.001 | 0.251 |

Abbreviations: MDD, major depressive disorder; HR, hazards ratio; CI, confidence interval; PM_2.5_, fine particulate matter with diameter ≤2.5μm; PM_10_, particulate matter with diameter ≤10μm; NO_2_, nitrogen dioxide; NO_X_, nitrogen oxides.

*P*-value for interaction: the interaction between air pollutions with age and gender.

All model was adjusted for age (stratified by gender), gender (stratified by age), ethnicity, education level, employment status, household income, and Townsend deprivation index.

**Table S15.** Association between genetic risk and major depressive disorder (MDD).

| Genetic Risk | HR (95%CI) for Continuous* | Low | Intermediate | High | *P* for trend |
| --- | --- | --- | --- | --- | --- |
|  |  | (n = 118,478) | (n =118,295) | (n = 118,124) |  |
| No. of MDD cases / person-years |  | 4,590/1,145,278 | 4,875/1,142,238 | 5,245/1,139,567 |  |
| Model 1 | 1.09 (1.07, 1.12) | 1.00 (Ref.) | 1.07 (1.02, 1.11) | 1.15 (1.10, 1.20) | <0.001 |
| Model 2 | 1.10 (1.07, 1.12) | 1.00 (Ref.) | 1.07 (1.03, 1.12) | 1.16 (1.11, 1.21) | <0.001 |

Abbreviations: MDD, major depressive disorder; HR, hazards ratio; CI, confidence interval; Ref, reference.

*Hazards ratio (HR) per inter quartile range (IQR) change of genetic risk score.

*P* value for trend calculated treating the genetic risk score as a continuous variable.

Model 1: Unadjusted.

Model 2: Adjusted for age, gender, ethnicity, education level, employment status, household income, and Townsend deprivation index, and genotyping batch and first 10 principal components of ancestry.

**Table S16**. Association between lifestyle category and major depressive disorder (MDD).

| Lifestyle Category | HR (95%CI) for Continuous* | Most healthy | Moderately healthy | Least healthy | *P* for trend |
| --- | --- | --- | --- | --- | --- |
|  |  | (n = 152,734) | (n =117,865) | (n = 8,489) |  |
| No. of MDD cases / person-years |  | 4,859/1,490,904 | 5,086/1,133,486 | 582/78,790 |  |
| Model 1 | 1.43 (1.39, 1.48) | 1.00 (Ref.) | 1.38 (1.33, 1.43) | 2.28 (2.09, 2.48) | <0.001 |
| Model 2 | 1.37 (1.33, 1.42) | 1.00 (Ref.) | 1.35 (1.29, 1.40) | 1.98 (1.81, 2.16) | <0.001 |

Abbreviations: MDD, major depressive disorder; HR, hazards ratio; CI, confidence interval; Ref, reference.

*Hazards ratio (HR) per inter quartile range (IQR) change of lifestyle category.

*P* value for trend calculated treating the lifestyle category as a continuous variable.

Model 1: Unadjusted.

Model 2: Adjusted for age, gender, ethnicity, education level, employment status, household income, Townsend deprivation index, and MDD-PRS.

**Table S17**. Risk of major depressive disorder (MDD) according to lifestyle score.

| Lifestyle score | No. MDD cases /person-years | Model 1 | |  | Model 2 | |
| --- | --- | --- | --- | --- | --- | --- |
|  |  | HR (95% CI) | P Value |  | HR (95% CI) | *P* Value |
| 0 (Most healthy) | 643 / 214,451 | 1.00 (Ref.) |  |  | 1.00 (Ref.) |  |
| 1 | 1,738 / 557,064 | 1.04 (0.95, 1.14) | 0.386 |  | 1.03 (0.94, 1.13) | 0.482 |
| 2 | 2,478 / 719,390 | 1.15 (1.05, 1.25) | 0.002 |  | 1.14 (1.04, 1.24) | 0.004 |
| 3 | 2,435 / 597,902 | 1.36 (1.25, 1.48) | <0.001 |  | 1.35 (1.24, 1.47) | <0.001 |
| 4 | 1,676 / 367,247 | 1.53 (1.39, 1.67) | <0.001 |  | 1.49 (1.35, 1.63) | <0.001 |
| 5 | 975 /168,337 | 1.94 (1.75, 2.14) | <0.001 |  | 1.80 (1.62, 1.99) | <0.001 |
| 6 | 392 / 60,075 | 2.19 (1.93, 2.48) | <0.001 |  | 1.95 (1.72, 2.22) | <0.001 |
| 7 | 155 / 15,813 | 3.29 (2.76, 3.93) | <0.001 |  | 2.74 (2.30, 3.28) | <0.001 |
| ≥8 (Least healthy) | 35 / 2,902 | 4.07 (2.90, 5.72) | <0.001 |  | 3.02 (2.14, 4.25) | <0.001 |
| *P* value for trend |  | <0.001 |  |  | <0.001 |  |

Abbreviations: MDD, major depressive disorder; HR, hazards ratio; CI, confidence interval; Ref, reference.

*P* value for trend calculated treating the lifestyle score as a continuous variable.

Model 1: Unadjusted.

Model 2: Adjusted for age, gender, ethnicity, education level, employment status, household income, Townsend deprivation index, and MDD-PRS.

**Table S18**. Multivariate-adjusted cox proportional hazard ratios of major depressive disorder (MDD) associated with ambient air pollutants stratified by lifestyle factors.

| Lifestyle factors | PM_2.5_ | |  | PM_10_ | |  | NO_2_ | |  | NO_x_ | |
| --- | --- | --- | --- | --- | --- | --- | --- | --- | --- | --- | --- |
|  | HR (95%CI) | *P*-interaction |  | HR (95%CI) | *P*-interaction |  | HR (95%CI) | *P*-interaction |  | HR (95%CI) | *P*-interaction |
| Smoking status |  |  |  |  |  |  |  |  |  |  |  |
| Healthy | 1.15 (1.05, 1.26) | 0.660 |  | 0.99 (0.90, 1.09) | 0.781 |  | 0.99 (0.96, 1.02) | 0.709 |  | 1.02 (1.00, 1.05) | 0.487 |
| Unhealthy | 1.09 (0.91, 1.32) |  |  | 0.96 (0.99, 1.00) |  |  | 1.00 (0.95, 1.06) |  |  | 1.00 (0.95, 1.05) |  |
| Alcohol intake |  |  |  |  |  |  |  |  |  |  |  |
| Healthy | 1.13 (1.03, 1.24) | 0.384 |  | 1.02 (0.93, 1.13) | 0.221 |  | 1.00 (0.97, 1.02) | 0.848 |  | 1.02 (1.00, 1.05) | 0.618 |
| Unhealthy | 1.29 (1.06, 1.55) |  |  | 0.89 (0.73, 1.09) |  |  | 1.01 (0.96, 1.07) |  |  | 1.04 (0.99, 1.09) |  |
| Physical activity |  |  |  |  |  |  |  |  |  |  |  |
| Healthy | 1.15 (1.03, 1.29) | 0.521 |  | 0.91 (0.81, 1.02) | 0.270 |  | 0.99 (0.96, 1.02) | 0.807 |  | 1.02 (1.00, 1.05) | 0.678 |
| Unhealthy | 1.12 (0.93, 1.34) |  |  | 1.07 (0.88, 1.31) |  |  | 0.99 (0.94, 1.05) |  |  | 1.02 (0.97, 1.07) |  |
| TV viewing time |  |  |  |  |  |  |  |  |  |  |  |
| Healthy | 1.13 (1.02, 1.25) | 0.422 |  | 1.00 (0.90, 1.12) | 0.739 |  | 1.00 (0.97, 1.03) | 0.996 |  | 1.02 (0.99, 1.05) | 0.940 |
| Unhealthy | 1.18 (1.03, 1.35) |  |  | 0.98 (0.85, 1.14) |  |  | 1.00 (0.96, 1.04) |  |  | 1.03 (0.99, 1.06) |  |
| Sleep duration |  |  |  |  |  |  |  |  |  |  |  |
| Healthy | 1.20 (1.08, 1.33) | 0.921 |  | 0.96 (0.86, 1.07) | 0.270 |  | 1.01 (0.98, 1.04) | 0.570 |  | 1.03 (1.00, 1.06) | 0.741 |
| Unhealthy | 1.08 (0.94, 1.24) |  |  | 1.05 (0.90, 1.21) |  |  | 0.98 (0.94, 1.02) |  |  | 1.01 (0.97, 1.05) |  |
| Fruit and vegetable intake |  |  |  |  |  |  |  |  |  |  |  |
| Healthy | 1.16 (1.06, 1.28) | 0.430 |  | 0.99 (0.90, 1.10) | 0.955 |  | 1.00 (0.97, 1.03) | 0.882 |  | 1.02 (1.00, 1.05) | 0.740 |
| Unhealthy | 1.07 (0.90, 1.28) |  |  | 0.96 (0.79, 1.16) |  |  | 0.97 (0.92, 1.03) |  |  | 1.01 (0.96, 1.06) |  |
| Oily fish intake |  |  |  |  |  |  |  |  |  |  |  |
| Healthy | 1.24 (1.11, 1.39) | 0.794 |  | 1.05 (0.93, 1.18) | 0.603 |  | 1.03 (0.99, 1.06) | 0.405 |  | 1.05 (1.02, 1.08) | 0.247 |
| Unhealthy | 1.08 (0.96, 1.22) |  |  | 0.95 (0.84, 1.09) |  |  | 0.97 (0.94, 1.00) |  |  | 0.99 (0.96, 1.03) |  |
| Red meat intake |  |  |  |  |  |  |  |  |  |  |  |
| Healthy | 1.08 (0.96, 1.21) | 0.089 |  | 0.96 (0.85, 1.08) | 0.456 |  | 0.98 (0.94, 1.01) | 0.059 |  | 1.01 (0.98, 1.04) | 0.140 |
| Unhealthy | 1.20 (1.06, 1.36) |  |  | 1.00 (0.88, 1.14) |  |  | 1.01 (0.97, 1.05) |  |  | 1.03 (1.00, 1.06) |  |
| Processed meat intake |  |  |  |  |  |  |  |  |  |  |  |
| Healthy | 1.10 (1.00, 1.22) | 0.022 |  | 0.95 (0.86, 1.06) | 0.081 |  | 0.99 (0.96, 1.02) | 0.140 |  | 1.02 (0.99, 1.04) | 0.154 |
| Unhealthy | 1.28 (1.11, 1.47) |  |  | 1.10 (0.94, 1.29) |  |  | 1.02 (0.97, 1.06) |  |  | 1.04 (1.00, 1.08) |  |

Abbreviations: MDD, major depressive disorder; HR, hazards ratio; CI, confidence interval; PM_2.5_, fine particulate matter with diameter ≤2.5μm; PM_10_, particulate matter with diameter ≤10μm; NO_2_, nitrogen dioxide; NO_X_, nitrogen oxides; Ref, reference.

*Hazards ratio (HR) per 5-μg/m^3^ change of PM_2.5_ level, per 10-μg/m^3^ change of PM_10_ and NO_2_ levels and per 20-μg/m^3^ change of NO_X_ level.

*P*-interaction was evaluated using hazard ratios for the product term between air pollutants and each lifestyle factors.

Model was adjusted for age, gender, ethnicity, education level, employment status, household income, and Townsend deprivation index.


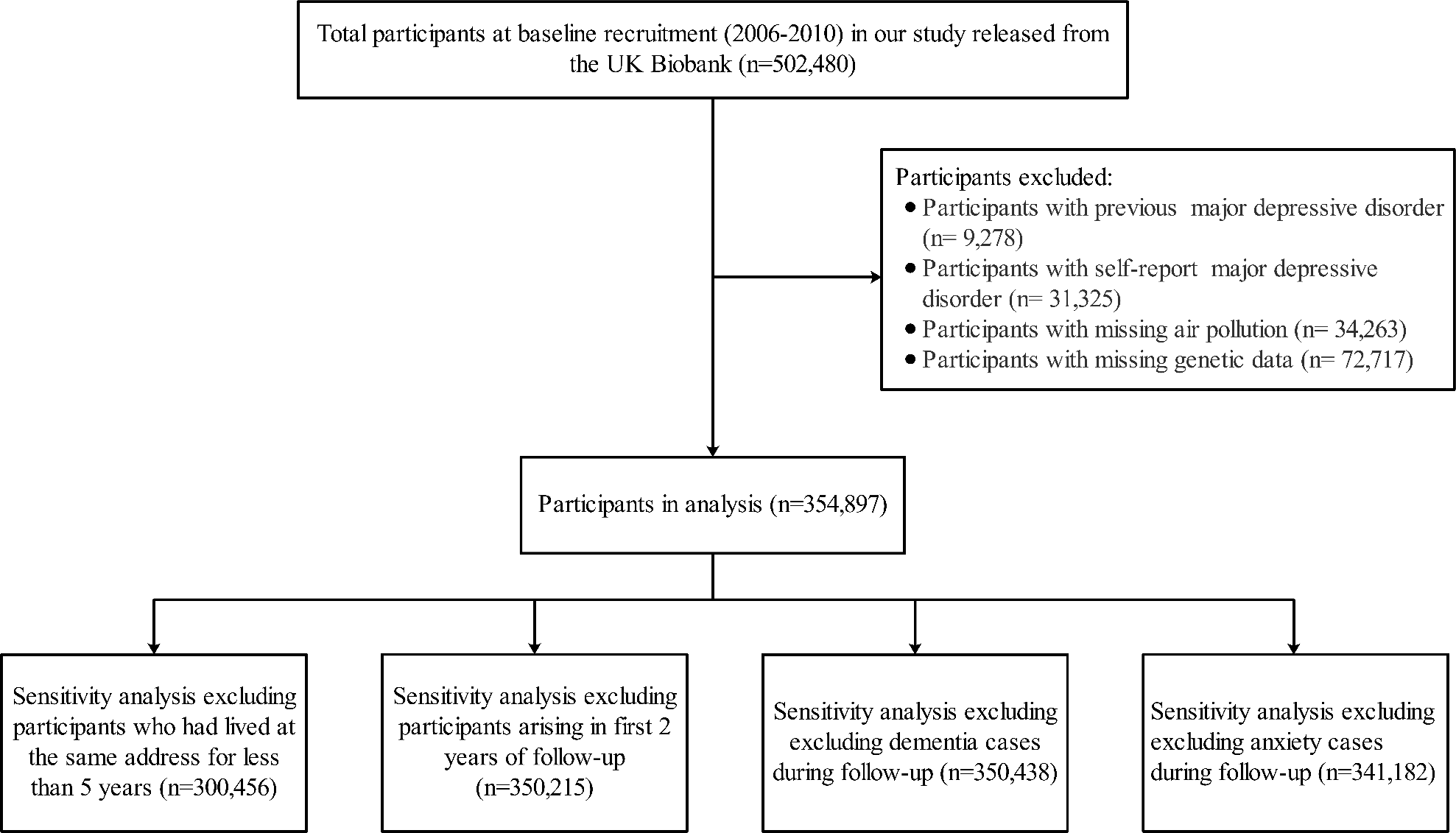


**Figure S1.** Flow of participants through study.


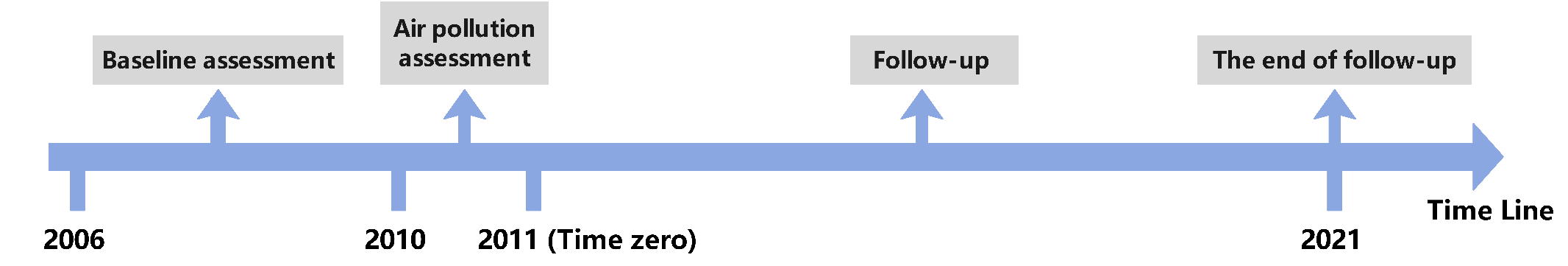


**Figure S2.** Summary about the time line of study design.

**
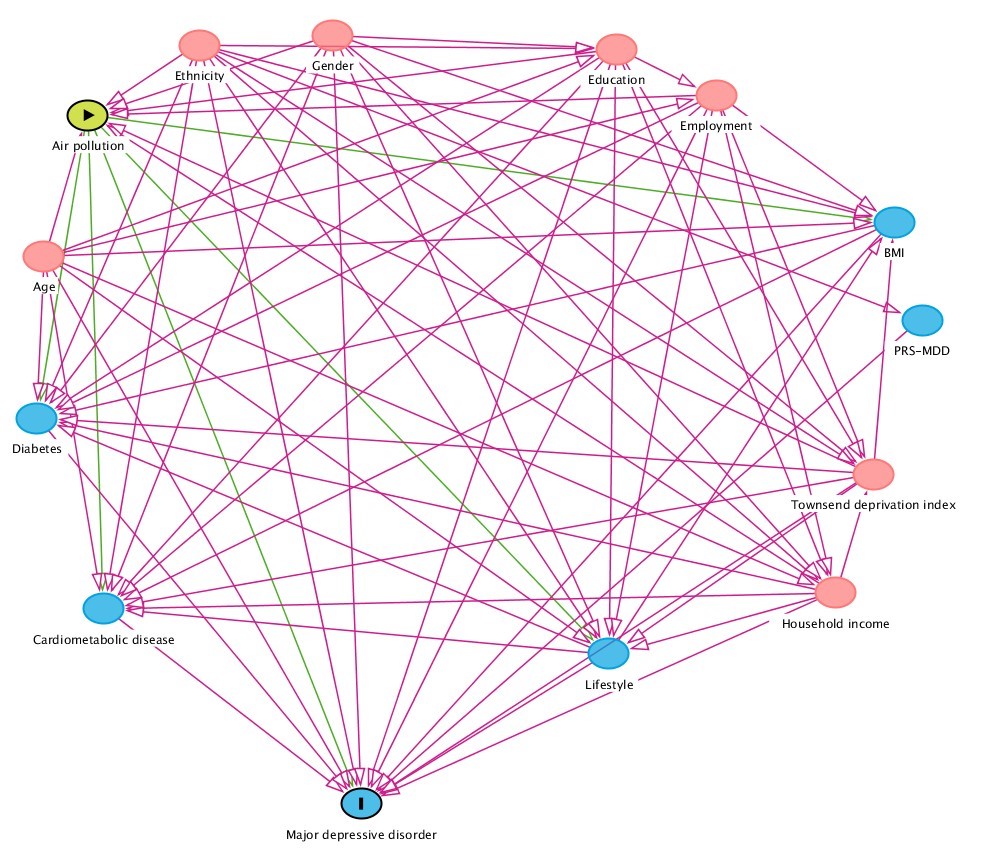
Figure S3.** Directed Acyclic Graph for the association between air pollution and major depressive disorder (MDD).


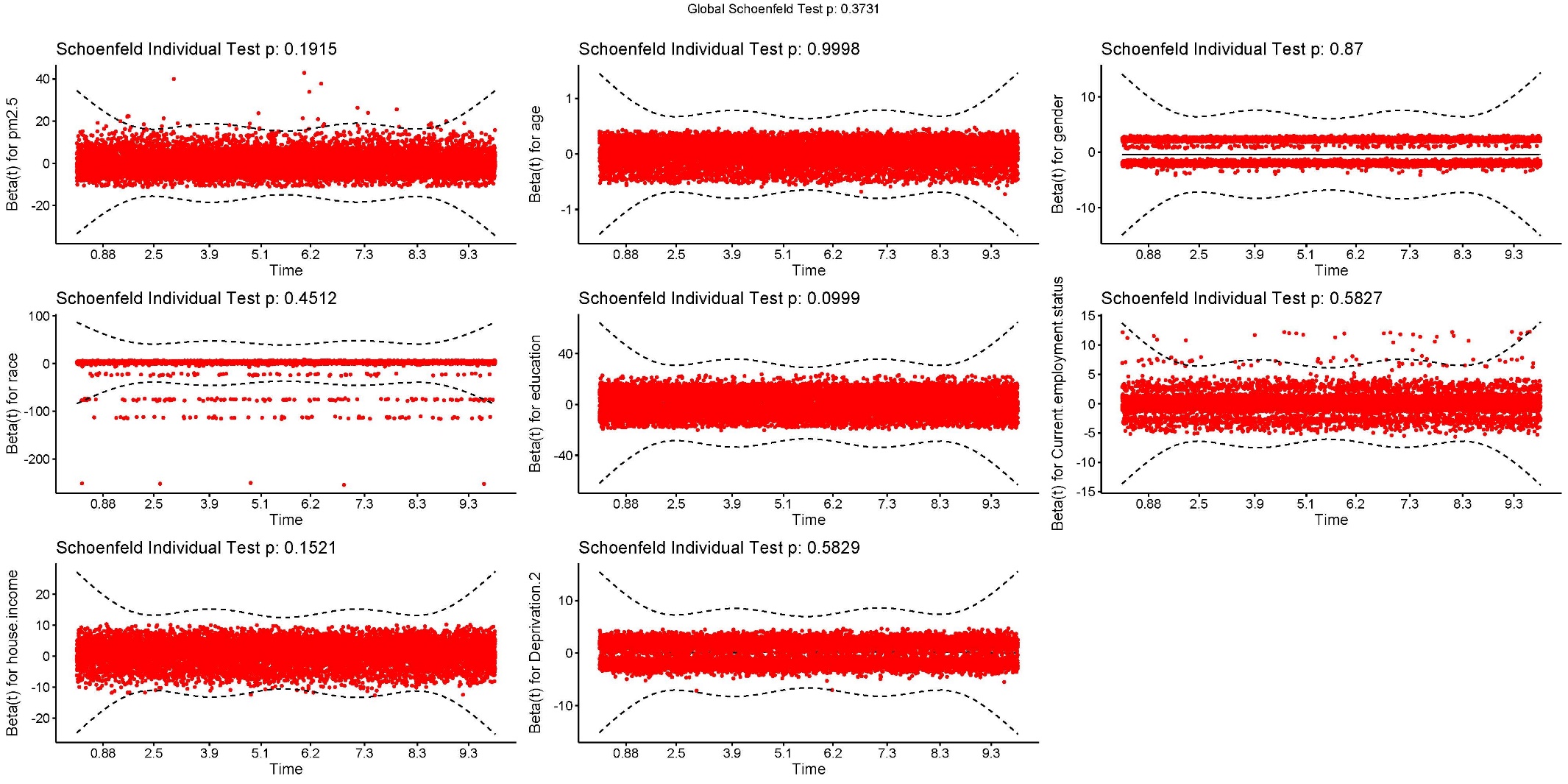


**Figure S4.** Schoenfeld residuals test of the Cox proportional hazards assumption based on PM_2.5_ and related covariates.


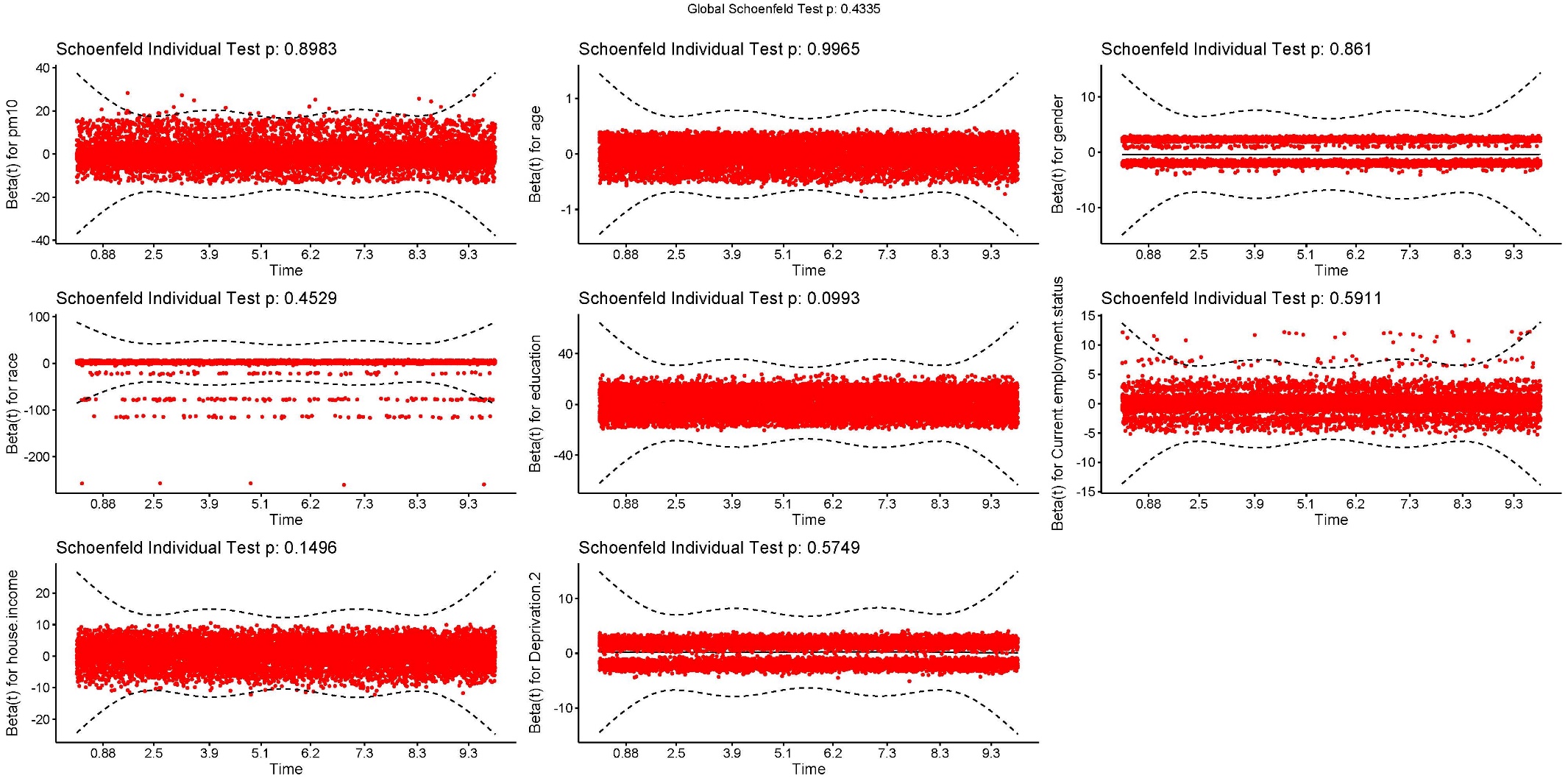


**Figure S5**. Schoenfeld residuals test of the Cox proportional hazards assumption based on PM_10_ and related covariates.


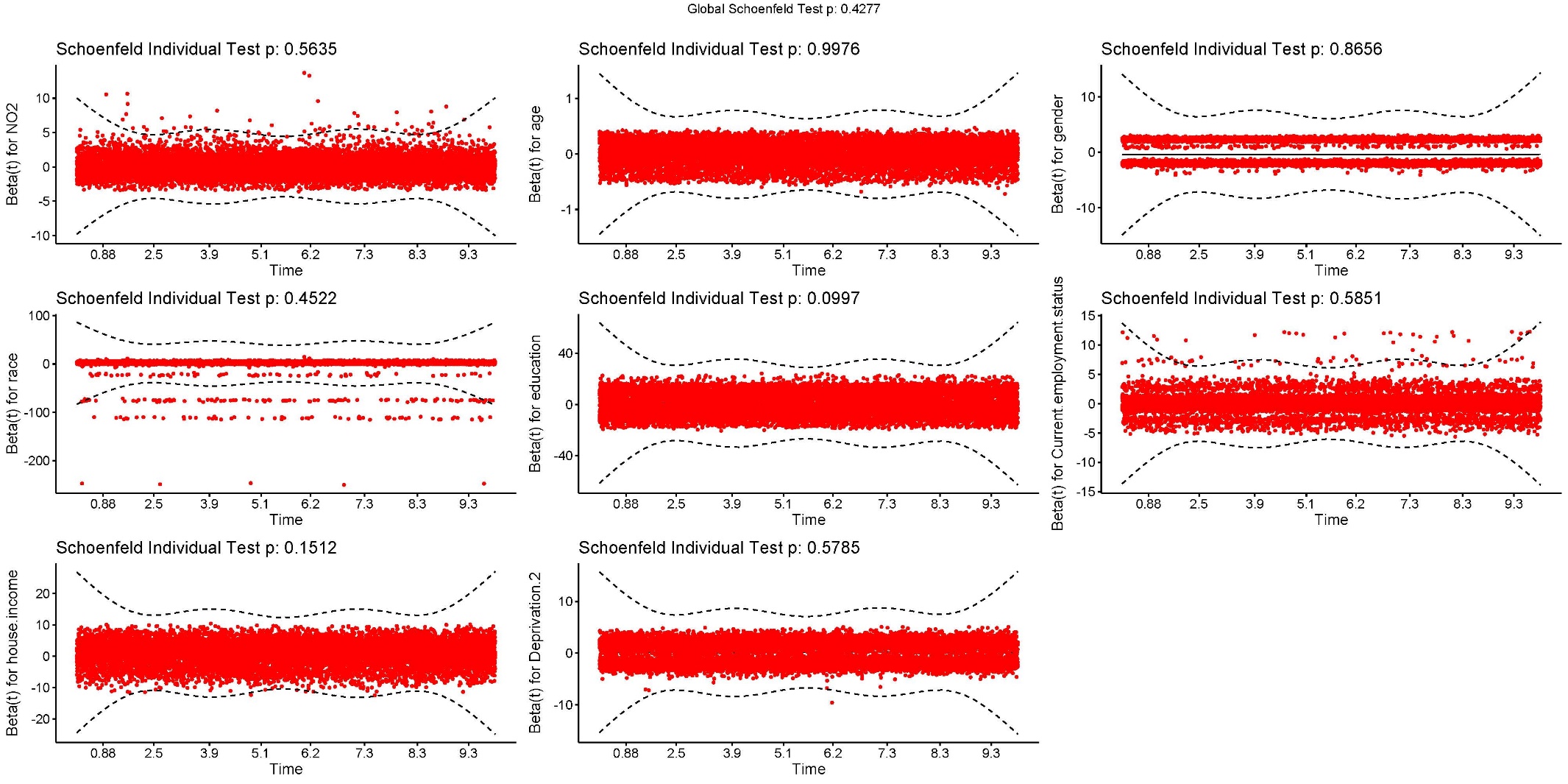


**Figure S6.** Schoenfeld residuals test of the Cox proportional hazards assumption based on NO_2_ and related covariates.


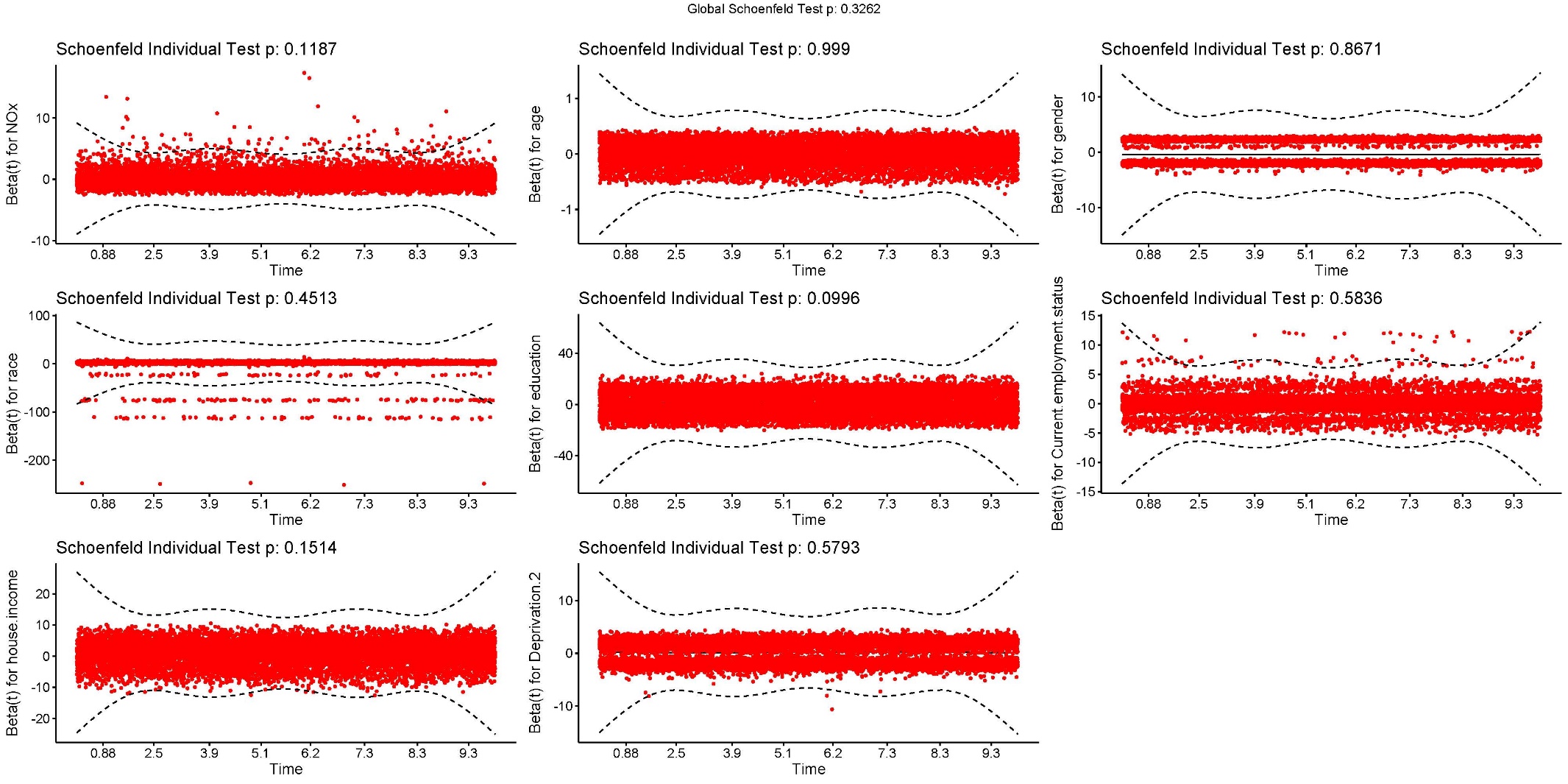


**Figure S7.** Schoenfeld residuals test of the Cox proportional hazards assumption based on NO_x_ and related covariates.


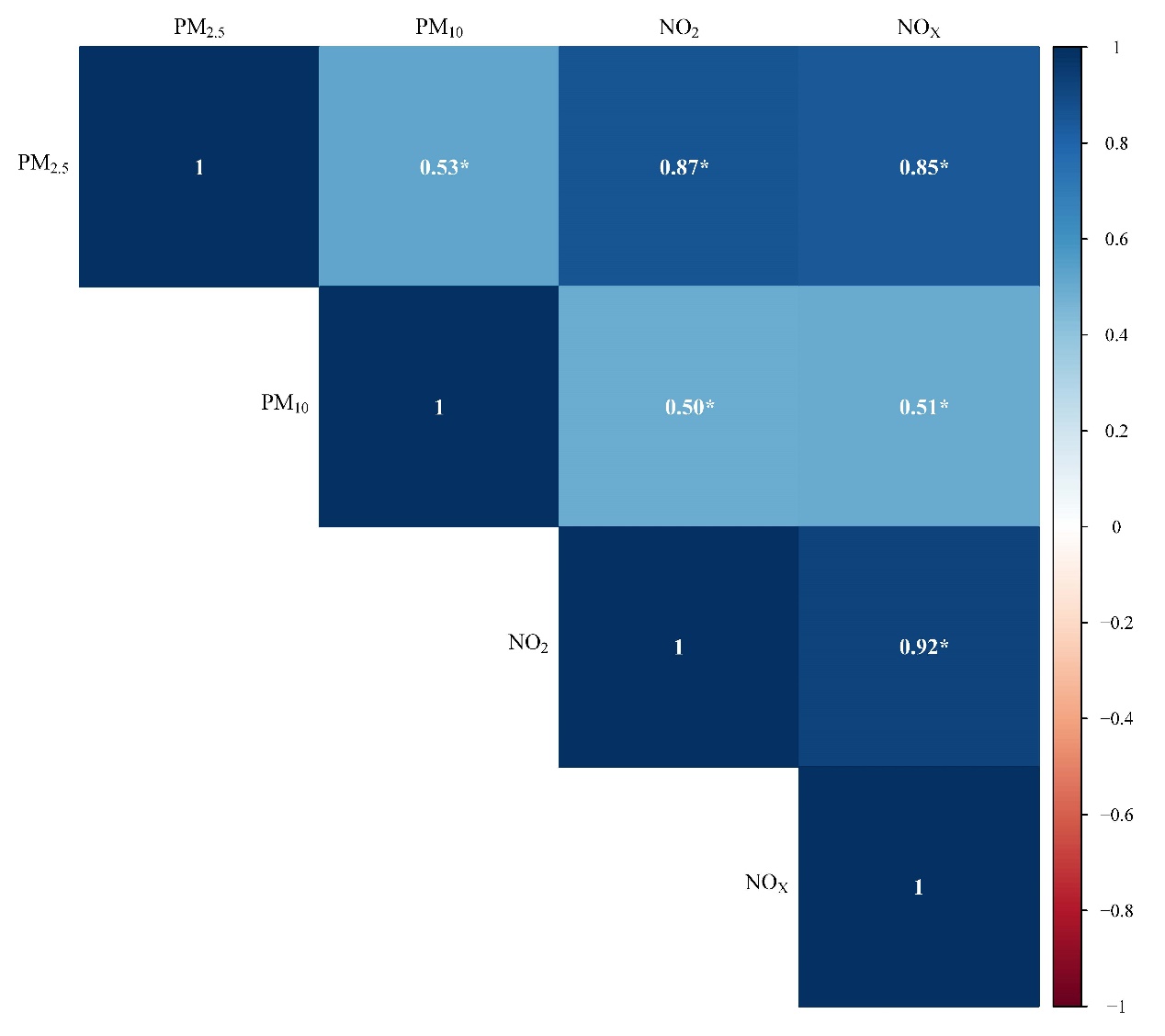


**Figure S8.** Pearson correlations between the individual air pollution incorporated in the study.

Abbreviations: PM_2.5_, fine particulate matter with diameter ≤ 2.5μm; PM_10_, particulate matter with diameter ≤ 10μm; NO_2_, nitrogen dioxide; NO_X_, nitrogen oxides.

Number indicates Pearson correlation coefficients.

*Indicates *P*-Value < 0.001.


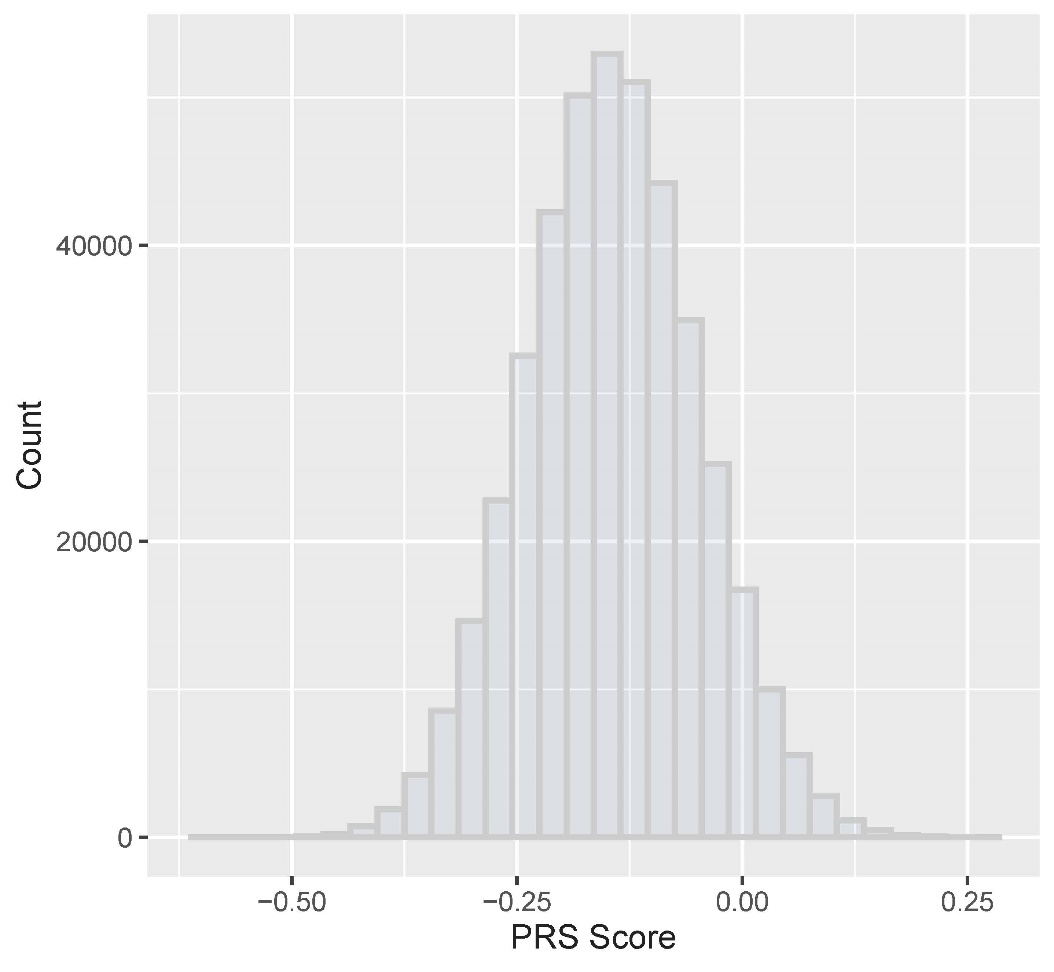


**Figure S9.** Distribution of major depressive disorder (MDD) genetic risk score.
